# Recombination and transposition drive genomic structural variation potentially impacting life history traits in a host-generalist fungal plant pathogen

**DOI:** 10.1186/s12915-025-02179-x

**Published:** 2025-04-28

**Authors:** Mark C. Derbyshire, Toby E. Newman, Yuphin Khentry, Pippa J. Michael, Sarita Jane Bennett, Ashmita Rijal Lamichhane, Carolyn Graham-Taylor, Subhash Chander, Claudia Camplone, Simone Vicini, Laura Esquivel-Garcia, Cathy Coutu, Dwayne Hegedus, John Clarkson, Kurt Lindbeck, Lars G. Kamphuis

**Affiliations:** 1 Centre for Crop and Disease Management, Curtin University, Perth, WA Australia; 2 Department of Genetics and Plant Breeding, Oilseeds Section, CCS Haryana Agricultural University, Hisar-125004, India; 3 Department of Agricultural, Food and Environmental Sciences, University of Perugia, Perugia, Italy; 4 Plant Science, Mcgill University, Sainte-Anne-de-Bellevue, QC Canada; 5 Agriculture and Agri-Food Canada, Saskatoon, SK Canada; 6 Warwick Crop Centre, School of Life Sciences, University of Warwick, Warwick, UK; 7 Department of Primary Industries, Wagga Wagga, New South Wales, Australia

**Keywords:** Recombination, Structural variant, Genome graph, Plant pathogen, Fungus, *Sclerotinia sclerotiorum*

## Abstract

**Background:**

An understanding of plant pathogen evolution is important for sustainable management of crop diseases. Plant pathogen populations must maintain adequate heritable phenotypic variability to survive. Polymorphisms ≥ 50 bp, known as structural variants (SVs), could contribute strongly to this variability by disrupting gene activities. SV acquisition is largely driven by mobile genetic elements called transposons, though a less appreciated source of SVs is erroneous meiotic double-strand break repair. The relative impacts of transposons and recombination on SV diversity and the overall contribution of SVs to phenotypic variability is elusive, especially in host generalists.

**Results:**

We use 25 high-quality genomes to create a graphical pan-genome of the globally distributed host-generalist crop pathogen *Sclerotinia sclerotiorum*. Outcrossing and recombination rates in this self-fertile species have been debated. Using bisulfite sequencing and short-read data from 190 strains, we show that *S. sclerotiorum* has many hallmarks of eukaryotic meiosis, including recombination hot and cold spots, centromeric and genic recombination suppression, and rapid linkage disequilibrium decay. Using a new statistic that captures average pairwise structural variation, we show that recombination and transposons make distinct contributions to SV diversity. Furthermore, despite only 5% of genes being dispensable, SVs often had a stronger impact than other variants across 14 life history traits measured in 103 distinct strains.

**Conclusions:**

Transposons and recombination make distinct contributions to SV diversity in *S. sclerotiorum*. Despite limited gene content diversity, SVs may strongly impact phenotypic variability. This sheds light on the genomic forces shaping adaptive flexibility in host generalists.

**Supplementary Information:**

The online version contains supplementary material available at 10.1186/s12915-025-02179-x.

## Background

An understanding of the evolutionary processes underpinning plant pathogen adaptation is crucial for developing better disease management strategies, such as resistant cultivars, prediction of epidemics and monitoring of fungicide resistance [1–3]. Population genetic approaches can be used to understand the evolutionary characteristics of plant pathogens [4, 5], although their application has been limited in the past to variants that can be confidently genotyped using short reads. However, with the now widespread use of long-read sequencing, more plant pathogen pan-genomes of increasing quality are becoming available for evolutionary studies [6–12].

Aside from simple genotypic variants, such as single-nucleotide polymorphisms (SNPs) and small insertions/deletions (InDels), complete genomes assembled using long reads can be used to identify structural variants (SVs), which are generally defined as polymorphisms of more than or equal to 50 bp [13]. These can be confidently genotyped using long-read assemblies and incorporated into a data structure known as a pan-genome graph [14, 15]. Based on this underlying representation of genomic variation, SVs can be genotyped in a broader set of individuals using short reads [16, 17]. This approach also improves the accuracy of non-SV calls by improving short-read placement and reducing reference bias [18].

In many species, this and similar techniques have revealed that previously invisible SVs are strongly linked with phenotypic variability. For instance, the tomato graph pan-genome showed that SVs are a major component of “missing heritability” [19], explaining much of the phenotypic variance not captured by simpler variants called against a single reference. Furthermore, in the fungal wheat pathogen *Zymoseptoria tritici*, SVs make a substantial contribution to important life history traits, such as fungicide tolerance [20].

Two key processes underpinning evolutionary adaptation are mutation, including de novo acquisition of SVs, and meiotic recombination. The traditional view is that mutation creates new alleles and meiotic recombination shuffles alleles to create new haplotypes [21]. Shuffling of alleles into novel haplotypes allows beneficial alleles to spread without the burden of linked deleterious alleles. Without meiotic exchange, populations are likely to gradually accumulate deleterious mutations that cannot be lost without also losing beneficial mutations, a process known as Muller’s ratchet [22–24].

Though evolutionary theory often ascribes distinct roles to mutation and meiotic recombination in creating and shuffling alleles, respectively, the two processes may not be completely independent. Meiosis itself may be powerfully mutagenic, as it requires the induction of numerous double-strand DNA breaks. Through erroneous repair of these breaks, meiosis has been linked with exceptionally high de novo mutation rates [12, 20, 21]. All types of mutations can occur through faulty repair of double-strand breaks, although meiosis-induced double-strand breaks may be particularly prone to creating new SVs [21].

In humans, for example, double-strand breaks induced by meiosis lead to a 400 to 1000-fold increase in SV acquisition rate, and many SVs induced in recombination hotspots are pathogenic, highlighting the impact of meiotic mutagenesis on phenotype and human disease [12, 25]. Recently, a machine learning approach showed that multiple genomic features, including local recombination rate, were highly predictive of SVs induced in haploid offspring of crosses of *Z. tritici* [20]. In the plant pathogen *Fusarium graminearum*, local recombination rate was also shown to be associated with SVs across four high-quality genomes [12].

In addition to meiosis, transposition is a highly potent instigator of structural variation in genomes. This occurs when active mobile elements called transposons duplicate or relocate themselves in the genome [26]. In addition, the repetitive nature of transposons can create SVs through pairing of distant genomic copies during DNA damage repair via the homologous recombination pathway [26]. Though transposons can be destabilising to genomes, occasionally they create beneficial mutations, which are an important source of adaptive evolution [27].

In plant pathogens, transposition is widely appreciated as one of the main driving forces of genomic plasticity. Though meiotic exchange has been linked with de novo acquisition of SVs in plant pathogenic fungi, the link between meiotic exchange and genome stability has not been widely explored in plant pathogen populations, and little is known about how meiosis and transposition interact to shape SV diversity. Despite several long-read pathogen pan-genomes, the overall contribution of SVs to variability in life history traits is also poorly understood.

To date, much of the research on the evolution of plant pathogen genomes has also been conducted on host specialists, which are under acute selective pressure to maintain virulence on a single species. In contrast, the fungus *Sclerotinia sclerotiorum* infects hundreds of plant species in at least 74 documented families [28]. Though its genome may harbour some polymorphic regions [29, 30], in contrast to many host specialists, its predicted effectors are largely conserved [30] and several are likely compatible with diverse hosts [31, 32]. This suggests that, like many niche-generalists, *S. sclerotiorum* has evolved an energetically optimised and multifunctional genome, which facilitates its colonisation of diverse hosts [33–35].

Sporulation in *S. sclerotiorum* occurs through obligate sexual reproduction. However, since it is self-fertile (homothallic), sexual reproduction can create genotypically uniform progeny, allowing certain genotypes to persist for long periods of time as clones [36]. The extent to which *S. sclerotiorum* outcrosses to generate new diversity has been debated, with some suggesting homothallism promotes universal outcrossing [29, 37–44] and others suggesting that outcrossing is extremely rare [45–47]. Consequently, the overall contribution of meiotic exchange to genome stability and evolution in this species is particularly poorly understood.

Here, we present a global graphical pan-genome of *S. sclerotiorum* and use 25 reference-quality genomes and 190 short-read samples to investigate species-wide SV diversity. To capture this diversity, we present a new statistic called “SVπ”, which describes the average number of SVs between all pairs of individuals. Using population genetics techniques, we establish *S. sclerotiorum* as an outcrossing species with many of the hallmarks of eukaryotic meiotic recombination, such as rapid linkage disequilibrium decay, suppressed recombination at centromeres, recombination hot and cold spots and enhanced recombination outside of coding sequences. We find that both recombination rate and transposable element content are independently positively correlated with total number of SVs and SVπ though not positively correlated with one another.

Overall, unlike that of most host specialists studied to date, we show that gene content in the *S. sclerotiorum* genome is largely stable, despite numerous small, unstable, repeat-rich, gene-sparse regions. SVs often had a stronger effect than other variants on 14 life history traits assessed across 103 strains, and we find that a 48 bp InDel is significantly associated with tolerance of the fungicide azoxystrobin. Overall, our data suggest that transposition and meiotic recombination make distinct contributions to SV diversity in *S. sclerotiorum*, and that SVs may be an important driver of phenotypic plasticity, despite the stability in gene content of the species. These insights shed new light on the genomic processes underpinning the evolution of host generalism in plant pathogens.

## Results and discussion

### Development of a *Sclerotinia sclerotiorum* graphical pan-genome

To create a high-quality set of *S. sclerotiorum* genomes for SV analysis, we generated Illumina-corrected Oxford Nanopore long reads assemblies of the genomes of 24 diverse strains from Australia (10 strains), Europe (5 strains) and Canada (9 strains). Overall, 23 of the strains had telomere-to-telomere assemblies for ≥ 10 of the 16 *S. sclerotiorum* chromosomes, and seven had telomere-to-telomere assemblies for ≥ 14 chromosomes. There were few gaps in assemblies on average, and 22 strains had gapless assemblies for ≥ 10 chromosomes; three of these strains had gapless assemblies for all 16 chromosomes. All these assemblies are comparable to the reference *S. sclerotiorum* genome [30], which has 14 telomere-to-telomere and 15 gapless chromosomes. BUSCO scores ranged from 98.9 to 99.2, with a median of 99.05 (Additional file 6: Supplementary Table 1), confirming the completeness of these assemblies. These new assemblies are available in NCBI under BioProject PRJNA1112094.

To explore structural variation in *S. sclerotiorum*, we constructed a pan-genome graph from the genomes of these 24 strains and the reference strain. In this graph, we identified 186,486 variants, including 154,892 SNPs, 5877 multiple nucleotide polymorphisms (MNPs), 20,061 InDels and 5556 SVs. There were 9876 complex variants with more than two alleles, including 2892 (52%) of the SVs.

To capture more genotypic diversity, we aligned Illumina short reads to the pan-genome graph from an additional 190 strains, 181 of which were sequenced in this study (available in NCBI under BioProject PRJNA1120954) (Additional file 7: Supplementary Table 2). Overall, the genotypes of 3741 of the SVs from the pan-genome graph were captured in this broader data set. This data set is the first graphical pan-genome of the important host generalist pathogen *S. sclerotiorum*. It includes 215 strains, with 152 from Australia, 17 from North America, 44 from Europe and one each from South Africa and Morocco.

### *Sclerotinia sclerotiorum* undergoes cryptic recombination whilst maintaining clonal lineages across large temporal and spatial distances

**Fig. 1:**
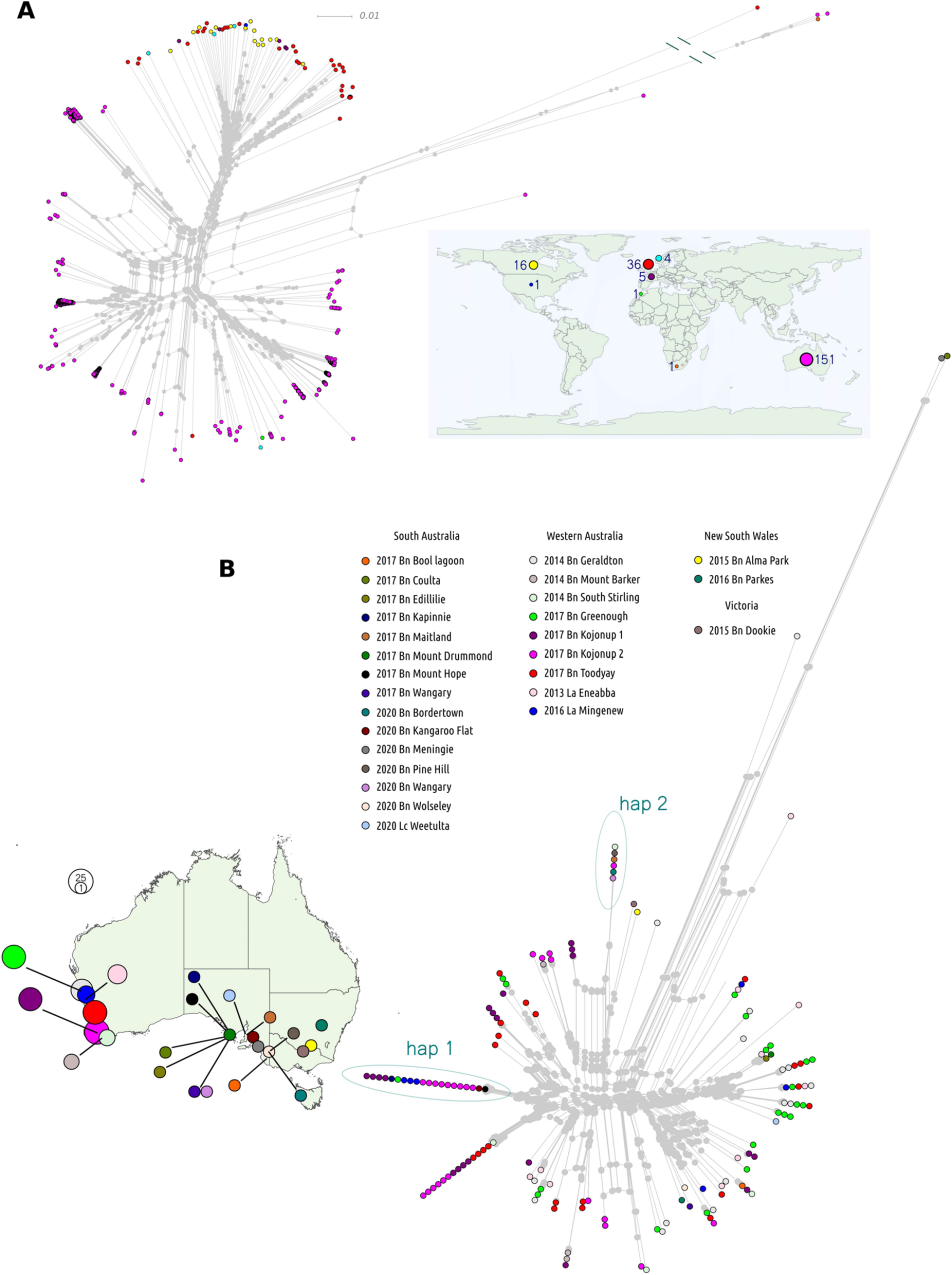
Genotypic clustering of *Sclerotinia sclerotiorum* strains from the global population sample.** A** A phylogenetic network with all strains in the dataset coloured according to geographical origin. The map inset shows where strains were collected with colours corresponding to those in the network. The sizes of circles on the map correspond with the number of strains from each global region. **B** A phylogenetic network for the Australian strains. Circles are coloured according to geographical origin within Australia. Where circles are stacked on top of each other, isolates are a group of clones. These were defined as groups of isolates where the two most distantly related individuals were at least 99.82% identical. The map to the left shows where isolates were collected within Australia, with colours of circles corresponding to colours on the network. The sizes of circles represent the numbers of strains from each collection site. Haplotypes 1 (hap 1) and 2 (hap 2) are examples of frequently sampled and geographically widespread clones, with individuals from Western Australia and South Australia

*S. sclerotiorum* produces ascospores through sexual reproduction. As it is homothallic, ascospores may be genotypically identical, which leads to an effectively clonal mode of propagation. Clonality is evident in the detection of temporally or spatially distant genotypically nearly identical strains. We identified 120 clonal lineages (in which the two most distantly related individuals were ≥ 99.82% identical) among the 215 strains (Additional file 1: Supplementary Fig. 1). Clonality was most prevalent among the Australian strains, whereas European and North American strains were mostly genotypically distinct (Fig. 1). This was expected because most of the European and North American strains were previously shown to be distinct lineages using markers [48, 49], whereas 99 of the Australian strains were collected from five sites (two of which were in the same locality) in Western Australia with no prior genotyping [50, 51].

Confirming the long-term maintenance of clonal propagation, we found several clones from geographically distant regions, some of which were collected many years apart. For example, strains CU11.18 and F19064 were found in Western Australia in 2013 and South Australia in 2018, respectively. The most extreme example was the pair of clones S55 and MB57, which were collected in the USA in 1987 and Manitoba in 2010, respectively.

In our previous study, we found that the global *S. sclerotiorum* population forms two distinct sub-populations, between which there has been limited gene flow. SNP data from the 215 genomes confirmed this observation (Fig. 1), showing that Australian/African and European/North American strains formed mostly distinct sub-populations (referred to as AuAf and EuNA herein). Although we expanded the Australian collection, our study only contained the two African strains from our previous study [29], Sssaf from South Africa and Ss44 from Morocco, so the global relationship between Australian and African strains is still not fully resolved.

**Fig. 2:**
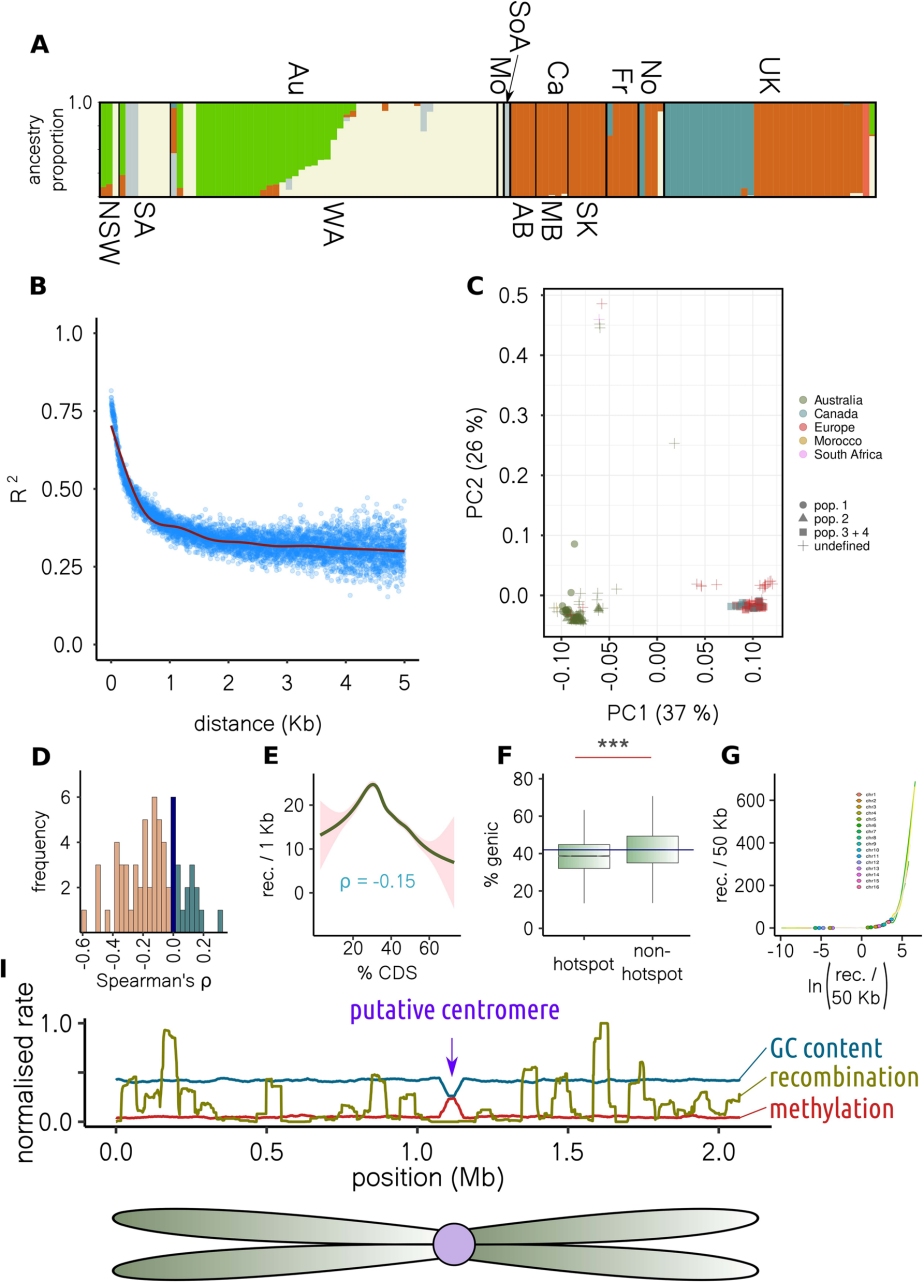
Population structure and evidence of recombination.** A** Colours correspond to ancestral populations making up individuals. Country of origin (above) is Au = Australia, Mo = Morocco, SoA = South Africa, Ca = Canada, Fr = France, No = Norway, and UK = UK. Below, states within Australia and Canada are indicated, where NSW = New South Wales, SA = South Australia, WA = Western Australia, AB = Alberta, MB = Manitoba, and SK = Saskatchewan. **B** Linkage disequilibrium (*y* axis) decay with physical distance (*x* axis). Points are averages for unique distance measurements, and the red line is a general additive model fit. **C** The first two principal components of genotypic variance. Colours indicate geographical origin and point shapes the four population sub-samples used for recombination analysis. **D** Across chromosomes and population sub-samples, the distribution of Spearman’s correlations between chromosome end distance and recombination rate. **E** Correlation between coding DNA sequence content (*x* axis) and recombination rate (*y* axis) of 50-kb sliding windows for population-4. The line is a general additive model fit. The same plots for all populations are shown in Supplementary Fig. 2. **F** Boxplot showing percent gene content of 50-kb windows containing and not containing recombination hotspots (*** = *P* < 2e^−16^). Boxes and whiskers show interquartile range. **G** Circles show where windows containing putative centromeres lie on a plot of recombination rate (*y* axis) against log recombination rate (*x* axis). Putative centromeres are in regions of low recombination, before the inflection point. **H** The *y* axis is scaled (division by maximum) recombination rate, amount of methylation or GC content for sliding windows. The *x* axis shows position (Mb) across chromosome 6 (all chromosomes and population samples are in Additional file 20: Supplementary File 1). All chromosomes had a dip in GC coincident with a spike in methylation, almost always coincident with a recombination cold spot

In the AuAf sub-population, we found evidence for three ancestral populations, and numerous admixed individuals. In the EuNA population, we identified three further ancestral populations with limited admixture (Fig. 2A). Two of the EuNA strains were admixed individuals containing alleles from either the AuAf ancestral populations or both the EuNA and AuAf ancestral populations. The widespread recent admixture among AuAf strains supports outcrossing between lineages from distinct ancestral populations.

To further explore outcrossing in the global *S. sclerotiorum* population, we investigated the rate of linkage disequilibrium decay in the 120 independent clonal lineages. We found that across the whole population, linkage disequilibrium decayed to half its maximum value at 428 bp (Fig. 2B). Three tests of the association between recombination and physical distance, neighbour similarity score [52], maximum Χ^2^ [53] and pairwise homoplasy index [54], also supported statistically significant recombination between non-adjacent alleles (*P* < 0.001 for all tests and chromosomes, Additional file 8: Supplementary Table 3).

The distance at which LD decays to half its maximum value (LD_2_) is typically smaller in predominantly outcrossing and larger in predominantly clonal species [55, 56]. Species that rarely outcross often have LD_2_ distances of more than 100 kb, whereas highly outcrossing species have LD_2_ distances on the order of a few hundred base pairs [56]. Though we have no direct assessment of the rate of outcrossing in *S. sclerotiorum*, the very small LD_2_ distance we observed suggests that it may be relatively frequent.

We used four unstructured population samples, referred to as population-1, population-2, population-3, and population-4, to calculate recombination rate across the genome. These contained 22, 13, 15 and 34 non-clonal isolates, respectively. The individuals in population-4 were from Canada and Europe, and those in population-3 were just the Canadian individuals from population-4. We chose to focus on both a combination of Canadian and European individuals with the same ancestry (population-4) to increase statistical power, and just the Canadian individuals (population-3) in case there were unaccounted for population differences due to geographic separation that might impact recombination rate estimation. The four population subsamples are indicated in the principal component analysis (PCA) plot in Fig. 2C.

Like other outcrossing species, recombination was not uniform across the genome. For all populations, there was a negative correlation between recombination rate and distance from chromosome ends in most chromosomes (Additional file 9: Supplementary Table 4). Overall, 12, 10, 11 and 10 out of 16 chromosomes displayed a significant negative correlation, for population-1, population-2, population-3 and population-4, respectively. There were two chromosomes with a significant positive correlation for population-1, population-2 and population-3, and four chromosomes for population-4. For all populations, the rest of the chromosomes did not show a significant correlation between recombination rate and distance from chromosome end. Figure 2D is a histogram of Spearman’s *ρ* values for recombination rate vs distance from chromosome end across all chromosomes and populations. It should be noted that the figure includes calculations across population-3 and population-4, which shared individuals.

Across the four population sub-samples, we identified 384 recombination hotspots (Additional file 9: Supplementary Table 4). Like other outcrossing species, we found that recombination rate was higher towards the ends of chromosomes where chromatin is more likely to be relaxed (Fig. 2D). Furthermore, recombination rate was negatively correlated with coding sequence density in all four populations, where Spearman’s *ρ* = − 0.15 for populations 1, 3 and 4, and − 0.13 for population-2 (*P* < 2e^−16^ for all populations). Though recombination rate was weakly negatively correlated with coding sequence density, the relationship between the two variables was not monotonic. Instead, there was an optimal gene density at which recombination rate peaked before declining rapidly (Fig. 2E, Additional file 2: Supplementary Fig. 2). We note that similar correlations may be expected for population-3 and population-4, which share individuals. However, a corresponding observation in the independent, genotypically distinct populations 1 and 2 provides further support for the weak relationship between recombination rate and gene density.

The union of recombination hotspots detected across all populations also had a lower gene density than other regions (*P* < 0.0001, Fig. 2F). We used the union because we aimed to detect the maximum number of hotspots present across the global meta-population of *S. sclerotiorum*. However, since population-3 and population-4 shared individuals, we also assessed the difference between hotspot and non-hotspot windows for each population individually. Regardless of population, windows containing recombination hotspots had a lower average gene density than others, although the difference was not significant for population-1 (*P* = 0.11). The largest population sample, population-4, exhibited a decrease in gene density from 42 to 40% in windows containing recombination hotspots (*P* = 5.49e^−43^) (Additional file 10: Supplementary Table 5). Based on a non-parametric randomisation test (see methods), the probability of random loci of equivalent sizes to the hotspots having as a low a gene density was < 0.001 for the union and populations 1, 3 and 4, and < 0.005 for population-2.

Our data suggest that recombination rate is generally higher outside of genic regions but low in the most gene-sparse parts of the genome. This is consistent with observations in other outcrossing species [57] where meiotic recombination within genes is selected against as it can lead to polymorphisms due to erroneous double-strand break (DSB) repair, though meiosis is repressed in the most gene-sparse regions, which also tend to be heterochromatic.

Across most chromosomes and all four population samples, there were clear recombination cold-spots that coincided with a single prominent drop in GC content and a single prominent spike in cytosine methylation based on bisulfite sequencing data generated in this study (Fig. 2G,H, Additional file 20: Supplementary File 1, Additional file 10: Supplementary Table 5). Decreased GC content and increased cytosine methylation are both hallmarks of eukaryotic centromeres [58, 59], around which meiotic recombination is typically suppressed [60]. The convergence of these three observations, and previous predictions from optical mapping data [61], suggest that these sites are the centromeres of the *S. sclerotiorum* chromosomes and, as in other outcrossing species, meiotic recombination is suppressed around them.

With the rapid decay of linkage disequilibrium, the presence of recombination hotspots, and the conspicuous recombination-related features characteristic of eukaryotic meiosis, we infer that *S. sclerotiorum* maintains genetic diversity across numerous populations through sexual outcrossing. Whilst clonal lineages may endure over extended periods via self-fertilisation, the ongoing process of sexual recombination among these lineages may be important for creating genotypic diversity. Presently, meiotic exchange is cryptic, as laboratory observations of sexual outcrossing are, to our knowledge, lacking.

Ecological theory suggests that loss of sexual reproduction initiates the gradual accumulation of deleterious alleles inseparable from beneficial ones, a phenomenon known as “Muller’s ratchet”. Consequently, strictly clonal populations are rare, with most facing a trajectory towards extinction. Given the continuing pressure on *S. sclerotiorum* for survival across numerous host species, coupled with its apparent lack of host preference, it is not surprising that it exhibits many attributes indicative of sexual outcrossing. Drawing from our findings and those of others [37, 43, 44], we suggest that homothallism in *S. sclerotiorum* not only supports persistence of certain clonal lineages but also fosters universal sexual compatibility.

### The *Sclerotinia sclerotiorum* pan-genome graph suggests transposable elements create hotspots of structural diversity

**Fig. 3:**
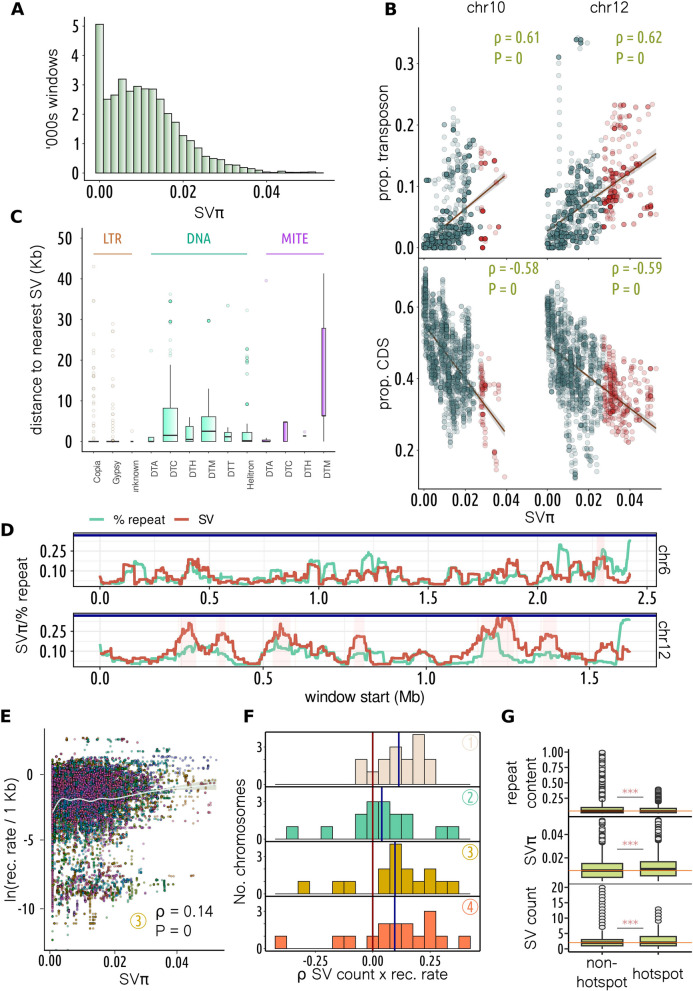
Analysis of structural variation across the *Sclerotinia sclerotiorum* pan-genome.** A** Distribution (*y* axis) of SVπ (x axis) for 50-kb sliding windows. **B** For chromosomes 10 and 12, correlation between SVπ (x axis) and proportion transposon (top *y* axis) or coding DNA sequence (bottom y axis). Spearman’s *ρ* and *P* value depicted top-right. Blue lines show linear regression of *y* onto *x* and the shaded area 95% confidence interval. Red points are SVπ hotspot (> 95th percentile) windows. **C** The *y* axis shows distance to nearest structural variant (SV) for transposon families. Transposon classification is indicated at the top and family on the *x* axis. Boxes and whiskers show interquartile range. LTR retrotransposons were generally closer than other transposons to SVs (Kruskal–Wallis *P* < 0.001; Dunn’s post hoc test shown in Additional file 11: Supplementary Table 6). **D** The *y* axis is SVπ or percent repeat for 50-kb windows (scaled for visualisation). The *x* axis shows window start (Mb), and plots show chromosomes 6 and 12, the latter having the highest average SVπ and the most hotspots (shaded in pink). **E** Correlation between log recombination rate per kb (*y* axis) and SVπ (*x* axis) across 50-kb sliding windows. Chromosomes are plotted in different colours and data shown are for population-3. Spearman’s *ρ* was between 0.14 and 0.15 for each of the population samples (*P* < 2e^−16^) but varied between chromosomes. **F** Distribution across chromosomes (*y* axis) of Spearman’s *ρ* for number of SVs and recombination rate in 50-kb sliding windows. Though correlation strength varied between chromosomes, correlations were generally positive. Colours and numbers correspond to the four population samples. **G** The *y* axis shows repeat content (top), SVπ (middle) and number of SVs (bottom) for windows that did not (left) and did (right) contain recombination hotspots. Boxes and whiskers show interquartile range; differences were significant according to a *t*-test (*** = *P* < 2.2e.^−16^)

To capture diversity of structural variation across the genome, we developed a statistic called “SVπ”. Akin to nucleotide [62] and synteny diversity [63], this statistic captures the average number of SVs per kb between all pairs of individuals. We found that SVπ was positively skewed when calculated for 50-kb sliding windows across the genome (Fig. 3A). This suggests that the *S. sclerotiorum* genome is mostly stable, with a few regions of excessive structural variation. We defined SV hotspots as sliding windows with a SVπ value above the 95th percentile across the genome. Interestingly, more hotspots were detected on some chromosomes than others. For example, chromosome 12 contained six hotspots and had an average SVπ of 0.016, whereas, despite being larger, chromosome 6 contained only one hotspot and had an average SVπ of 0.009 (Fig. 3D, Additional file 3: Supplementary Fig. 3).

Transposable element and gene content were positively (Spearman’s *ρ* = 0.28, *P* < 2e^−16^) and negatively (Spearman’s *ρ* = − 0.33, *P* < 2e^−16^) correlated with SVπ, respectively. The correlation between transposon/gene content and SVπ varied between chromosomes, with the highest correlations observed on chromosomes 10 and 12 (Spearman’s *ρ* = 0.61 and 0.62, Spearman’s *ρ* = − 0.58 and − 0.59, respectively (*P* < 2e^−16^)) (Fig. 3B).

The association between SVs and transposable elements was further supported by the observation that SVs were significantly closer than randomised loci to the nearest transposon across all genomes (*P* < 0.0001). Transposable elements in the long terminal repeat (LTR) family were significantly (*P* < 0.0001) closer to the nearest SV than those in eight out of 10 other families (Fig. 3C, Additional file 11: Supplementary Table 6), suggesting they may strongly contribute to genome instability in *S. sclerotiorum*.

LTRs are a type of retrotransposon, which are transposons characterised by a copy and paste proliferation mechanism that involves transcription into RNA, reverse transcription into DNA and re-insertion into the genome [64]. Retrotransposons are unique to eukaryotes [65], and their replicative ability has made them often the dominant transposon class in eukaryote genomes [66]. Several studies have linked retrotransposons with virulence evolution in plant pathogens [67, 68], including the host generalist species *Botrytis cinerea*, where they have been shown to encode small RNA effectors [69]. Our observations that retrotransposons are most strongly linked of all transposon classes to SVs suggest that they are the most active mobile elements in *S. sclerotiorum*. Their ongoing contribution to structural variation may be important for genomic plasticity in this species.

Stable, gene-dense and repeat-poor, and unstable, gene-sparse and repeat-rich genomic regions are common across eukaryote genomes [70]. The accumulation of transposons and SVs in gene-sparse regions is likely a result of relaxed selective pressure and accumulation of largely selectively neutral alleles. These regions can be important for adaptive evolution because they harbour extensive diversity in gene content and gene sequences [71]. When the environment changes, previously selectively neutral mutations may confer an advantage, leading to ongoing maintenance of these regions, and the transposons within them, in populations [72]. Our data show that, like those of most eukaryotes, the *S. sclerotiorum* genome is also partitioned into stable and unstable regions, and unstable regions are likely most strongly shaped by LTR retrotransposons. Overall, transposon content in the 25 *S. sclerotiorum* genomes was relatively low at 5.51 to 6.91% (Additional file 12: Supplementary Table 7). Despite this, transposable elements are strongly linked to diversity in SVs across the *S. sclerotiorum* genome.

### Recombination and transposable elements make distinct contributions to structural variation

Several studies have shown that besides transposition, structural variation can be caused by recombination. However, little is known about the overall impact of recombination on structural variation in natural populations. In *S. sclerotiorum*, we found an overall correlation between SVπ and recombination rate for all four population samples we used for recombination rate estimation (Fig. 3E, Spearman’s *ρ* = 0.14–0.15, *P* < 2e^−16^). Though this is suggestive of a link between SV diversity and recombination, it does not necessarily imply that recombination creates SVs, as this relationship could also be caused by increased haplotype diversity in regions with a high recombination rate. Therefore, to determine whether genomic regions with a high recombination rate may be more prone to development of SVs, we assessed the correlation between recombination rate and the overall number of SVs called against the reference genome. Though the strength of correlation between these parameters varied considerably between chromosomes and populations, we found that, on average, there was a weak to moderate correlation between total number of SVs and estimated recombination rate (Fig. 3F, mean Spearman’s *ρ* = 0.09). For 12, 8, 13, and 12 out of 16 chromosomes, for the four respective populations, there was a significant positive correlation between recombination rate and total number of SVs (*P* < 0.05, Additional file 13: Supplementary Table 8). In contrast, only 1–3 chromosomes displayed a significant negative correlation between recombination rate and number of SVs.

Despite the correlations between recombination rate and both SVπ and total SVs across chromosomes and populations, there were far fewer instances of a positive correlation between recombination rate and transposon content, and the overall average of all Spearman’s *ρ*s was close to zero at − 0.0018 (Additional file 13: Supplementary Table 8). Furthermore, based on the union of recombination hotspots across populations, we found that windows containing them had a slightly but significantly lower average transposable element content than others (5.71% vs 6.97%, *P* < 2.2e − 16), despite having elevated SVπ (average of 0.012 vs 0.010, *P* < 2.2e − 16) and more SVs (2.65 vs 2.40, *P* < 2.2e − 16) (Fig. 3G). Though a non-parametric randomisation test did not support a lower than random repeat content for hotspot windows (*P* = 0.46), it supported an elevated SVπ and number of SVs (*P* = 0.012 and *P* = 0.001, respectively). Together, these analyses suggest that SVs are more likely to appear in genomic regions prone to meiotic recombination, despite these regions not being strongly (or possibly negatively) associated with transposable elements.

We found similar results when considering hotspots individually detected across populations 1, 2, 3 and 4, respectively, with repeat content decreasing from 6.5 to 5.8% (*P* = 1.59e^−13^), 6.5 to 6.3% (*P* = 0.046), 6.5 to 6.1% (*P* = 2.5e^−5^), and 6.7 to 5.7% (*P* = 5.78e^−36^); SVπ increasing from 0.011 to 0.013, 0.011 to 0.012, 0.011 to 0.012, and 0.010 to 0.012 (*P* < 0.0001); and SVs increasing from 2.45 to 2.96, 2.48 to 2.70, 2.49 to 2.62 and 2.44 to 2.70 (*P* < 0.001). Based on a non-parametric randomisation test, *P* values for populations 1, 2, 3 and 4, respectively, were 0.61, 0.85, 0.20 and 0.39 for decreased repeat density; 0.005, 0.076, 0.071 and 0.04 for SVπ; and 0.008, 0.224, 0.20 and 0.017 for number of SVs. As for the union of hotspot regions, this did not support an association between hotspot regions and repeat density. Additionally, the likelihood of elevated SV number and SVπ in hotspots varied between populations and was not any less likely than random for some. However, we suggest that the union of hotspot regions may provide the most reliable estimate, as it represents the highest sensitivity of detection. Furthermore, population-4, which had the largest sample size, supported an elevation of SVs and SVπ within hotspot regions.

This suggests that meiotic recombination and transposition make independent contributions to structural variation. In agreement, we found that the number of SVs was better described in a regression model by both average recombination rate and transposon content than transposon content alone, though transposon content was the dominant predictor in the model (likelihood ratio test *P* < 2.2e − 16, transposon *F* = 74.86, recombination rate *F* = 13.08).

Our analyses document an interesting link between estimated recombination rate and the rate of structural variation in the *S. sclerotiorum* genome. This is not surprising given the mutagenic properties of meiosis. Given the relatively low level of transposable element content in the *S. sclerotiorum* genome, recombination through meiotic exchange could be an additional important source of structural variation. Our regression model suggests that recombination rate is far outweighed by transposon density as a predictor of genome stability. However, since recombination rate was typically higher in regions of intermediate gene density, recombination may have a greater chance of inducing SVs that impact gene function.

### *Sclerotinia sclerotiorum* has a closed pan-genome with relatively few non-syntenic blocks of genes

**Fig. 4:**
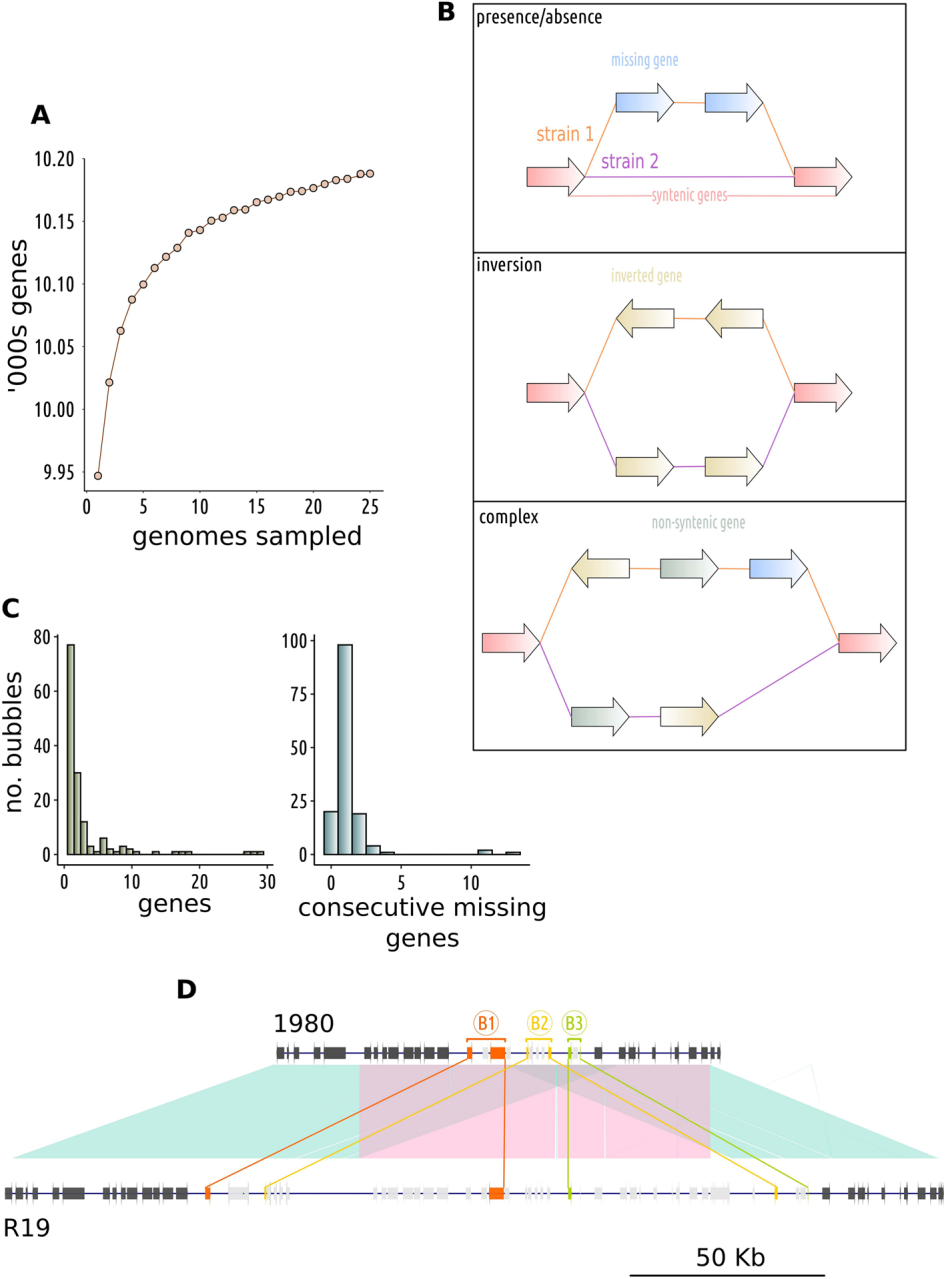
Gene content variability in the *Sclerotinia sclerotiorum* pan-genome.** A** The relationship between total number of unique genes (*y* axis) and number of genomes sampled (*x* axis). **B** An illustration of the types of variation gene bubbles might contain. **C** Number of gene bubbles (*y* axis) and number of genes they contained (top) or number of consecutive missing genes they contained (bottom). **D** A region in the 1980 reference genome that had a partial duplication in the isolate R19 and no other isolates. This region contained the largest three gene bubbles, indicated here with B1 (orange), B2 (yellow) and B3 (green). Start and end genes for each called bubble are indicated in their respective colours and non-syntenic genes within bubbles are in light grey. Neighbouring genes are in dark grey. The shaded area connects homologous regions and the pink region is duplicated in R19

The gene-space within a pan-genome lies on a spectrum from high variability in certain species to remarkable stability in others. Species harbouring a limited number of dispensable genes are characterised by closed pan-genomes, whilst those with diverse gene content are classified as having open pan-genomes [73]. To assess the openness of the *S. sclerotiorum* pan-genome, we sampled from two to all 25 strains in our dataset and plotted number of strains against number of novel genes. We found that the number of additional genes brought by adding a new strain plateaued quickly at 5–10 strains, indicating that most dispensable genes in the population are present in multiple strains (Fig. 4A).

Across all strains, we identified 10,188 unique genes, of which 553 (5.43%) were dispensable. Few informative Gene Ontology terms were over-represented among these genes, although we noted an over-representation of the term “GO:0031177” (*P* = 0.012), which is ascribed to genes containing a phosphopantetheine binding site. Since one of the main functions of this site is in secondary metabolite biosynthesis by non-ribosomal peptide synthases (NRPSs) and polyketide synthases (PKSs), it is not surprising that we also found an over-representation of genes in secondary metabolite biosynthesis clusters among dispensable genes (odds ratio = 2.28, *P* = 1.44e − 11). We found no significant over-representation of secreted proteins, regardless of size (odds ratio = 0.73, *P* = 0.18 for ≥ 300 amino acids; odds ratio = 1.38, *P* = 0.19 for ≤ 300 amino acids).

Based on a graphical representation of gene synteny, we identified 615 runs of one or more genes that were non-syntenic between strains. In keeping with graph terminology, we refer to these as “gene bubbles”. A gene bubble encompasses any break in gene synteny. The genes to the left and right of a bubble are present in all strains in the same orientation and order relative to one another. Between these two genes, any number of rearrangements or deletions may have occurred, disrupting the synteny of genes across strains. The three broad scenarios captured by gene bubbles are illustrated in Fig. 4B. Bubbles can contain “missing genes”, where the gene is not present in at least one strain within that bubble, and they may contain inverted or re-arranged genes, where the gene is present in all strains but has changed orientation or location relative to other genes. Complex bubbles can have a mixture of all three of these types of break in synteny. A consecutive run of missing genes is a run of adjacent genes missing in the same strain, which may represent a large insertion/deletion.

The number of genes in gene bubbles ranged from 1 to 34, with most gene bubbles containing only a single gene (Fig. 4C). Consecutive runs of missing genes within bubbles ranged from 0 (i.e. the bubble was an inversion) to 13 (median = 1) (Fig. 4C). The largest three consecutive runs of missing genes within bubbles were identified on chromosome 12, which was the chromosome with the highest SVπ. Closer inspection of these runs identified a complex region partially duplicated in the strain R19, which was sampled in 2007 from buttercup in Warwickshire in the UK (Fig. 4C). Many of the genes in this region were likely transposon genes, as they were annotated with Pfam domains such as RNase H (PF00075), reverse transcriptase (PF00078) and endonuclease (PF14529) (Additional file 14: Supplementary Table 9). However, there were also glycosyl hydrolases (PF00722), ubiquitins (PF00240) and a major facilitator superfamily transporter (PF07690). The fact that this region was the same in all strains apart from R19 could mean it is deleterious. Alternatively, it could be a relatively new polymorphism whose evolutionary fate has not yet been determined. So far, the polymorphism does not appear to be detrimental to infection on brassicas, since R19 is more aggressive than several other diverse isolates from the UK [74].

The closed *S. sclerotiorum* pan-genome contrasts the pan-genomes of host specialist fungal pathogens. For instance, in a population sample of 19 global isolates of *Z. tritici*, approximately 40% of genes were dispensable [8], and in 26 strains of the wheat pathogen *Pyrenophora tritici-repentis* 43% [75].

To our knowledge, little is known about what shapes pangenome openness in eukaryotes. However, ecological theory suggests that selective pressure from the host is stronger on host specialists than generalists [33]. To our knowledge, there are no *S. sclerotiorum* strains unable to reproduce on a single host species or genotype. It is unlikely, therefore, that a single virulence gene, such as an effector, would ever confer a strong host-driven selective advantage in this species. Therefore, maintenance of a repertoire of dispensable virulence proteins to ensure adaptability to a constantly changing host environment seems unlikely. Instead, the closed pan-genome of *S. sclerotiorum* aligns with previous research suggesting that it and other host generalists have evolved towards energetic optimisation of core virulence genes that function on multiple host species [31, 34].

### Structural variation may have a strong impact on adaptive flexibility of life history traits

Adaptive flexibility and fitness of a population are underpinned by genotypic variation that impacts life history traits. As a global host generalist agricultural pest, *S. sclerotiorum* is exposed to diverse environments and must be adaptable to a range of temperatures and stressors, such as host metabolites. To assess global phenotypic diversity in *S. sclerotiorum*, we measured 14 life history traits across 103 genotypically distinct strains, including relative growth on the Brassicaceae defence compounds brassinin and camalexin, the Fabaceae defence compound medicarpin, the reactive oxygen species H_2_O_2_ (ROS), and the two fungicides azoxystrobin and tebuconazole; growth and relative growth at 15, 20 and 25 °C; and fecundity-related traits including number, and average and total weight of sclerotia.

**Fig. 5:**
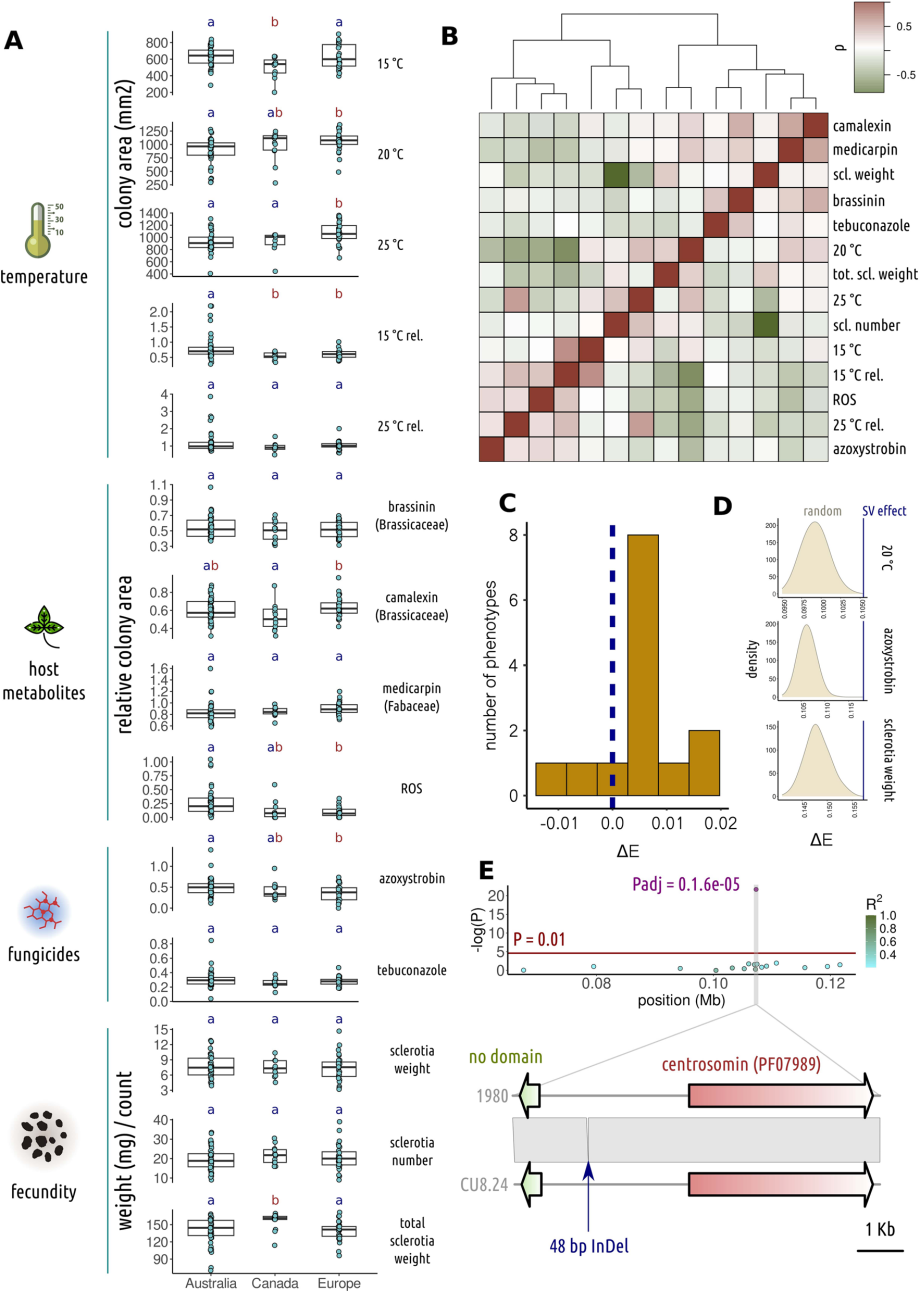
Life history traits assessed across a subset of *Sclerotinia sclerotiorum* strains.** A** Boxplots of measurements for life history traits (indicated to the right of the plot) in four categories (to the left). The distribution is plot for strains from the three major geographical regions, Australia, Canada and Europe. Points are the individual data points and box and whisker plots show interquartile range. The letters *a* and *b* above plots indicate significant differences between groups. **B** The top panel is a heatmap (rows are in the same order as columns), showing Pearson’s *ρ* between measurements for the 14 life history traits. Colouring goes from green (negative correlation) to red (positive). The dendrogram shows hierarchical clustering of the traits. **C** The distribution (*y* axis) across the 14 traits of mean effect size of non-SVs subtracted from mean effect size of SVs (ΔE), where effect sizes are absolute. **D** The *y* axis shows the density of measurements of absolute effect size for 500 random samples with an identical minor allele frequency distribution to that of SVs. The blue line shows the observed absolute effect size for SVs. For these three traits, the *P* value of this test was < 0.002. **E** A region surrounding a quantitative trait locus (QTL) for relative rate of growth on azoxystrobin (top), with − log(P) on the *y* axis and position in Mb on the *x*. The colours of points represent linkage disequilibrium of variants at the different positions with the QTL (the purple point with associated *P* value). The red line is a *P* value of 0.01. Below this region, the two genes neighbouring the 48 bp InDel underlying the QTL are illustrated. These included a small gene with no known domains and a larger centrosomin-encoding gene

We found significant differences between isolates from different geographical origins for eight of these traits. Both European and Australian strains grew faster at 15 °C than Canadian strains (Fig. 5A) (*P* = 0.014 and 0.007, respectively). At 20 °C, European strains grew significantly faster than Australian but not Canadian strains (*P* = 0.003 and 0.52, respectively), though Canadian strains grew at a similar rate to Australian strains (*P* = 0.40). At 25 °C, European strains grew faster than both Canadian and Australian strains (*P* = 0.035 and 0.00072, respectively). Relative growth (growth divided by growth at 20 °C, generally considered the middle of the optimum range [76, 77]) at 15 °C was significantly lower for both European and Canadian strains compared with Australian strains (*P* = 0.035 and 0.049, respectively), though relative growth at 25 °C was not significantly different between strains from different global regions.

Differences in growth rate at different temperatures between these populations could be a result of adaptation to prevailing temperatures during the growing season for major host crops, a phenomenon that has been previously observed at a local level in Australia [50, 51, 78]. However, it is difficult to completely align our observations with the likely reproductive cycle of *S. sclerotiorum* in these three global cropping regions, as different host crops are likely to be available at different times of year. For example, the major host species *Brassica napus* usually flowers in spring in the UK, where temperatures are often lower than when *B. napus* flowers in Western Australia in July. On the other hand, some hosts, such as lettuce, may be also present later in the season in the UK. A weaker adaptation to lower temperatures is possible for Canadian strains, which would likely infect *B. napus* when it is flowering during the hotter summer months.

Among host antimicrobial metabolites, we found in European strains a significant increase in growth on camalexin compared with Canadian strains (*P* = 0.015) and a significant decrease on ROS (0.0015) compared with Australian strains. Similarly, European strains had a significantly lower growth rate on azoxystrobin compared with Australian strains (*P* = 0.035), and Canadian strains were in the middle (*P* = 0.69 and 0.64 compared with Australian and European strains, respectively).

Interestingly, Canadian strains produced a greater total sclerotia weight compared with strains from Australia and Europe (*P* = 0.034 and 0.036, respectively). This seemed to be due to a slight increase in the mean of both sclerotia number and weight. The size of sclerotia has been previously linked with the rate of germination [79] and number of apothecia per sclerotium [80], suggesting it is an important component of fecundity. Phenotypic variation in this trait may have important implications for pathogen proliferation and epidemic potential of different populations.

The traits we measured had complex genetic synergisms and antagonisms with one another, for instance brassinin and camalexin tolerance were positively correlated (Pearson’s *ρ* = 0.43) and shared positive genetic covariance (0.93 (SE ± 0.16)) (Fig. 5B, Additional file 6: Additional file 15: Supplementary Table 10). The same was true of camalexin and medicarpin tolerance (Pearson’s *ρ* = 0.48, genetic covariance = 0.65 (SE ± 0.35)). Other traits were negatively correlated and had negative genetic covariance, for instance growth at 20 °C and azoxystrobin tolerance (Pearson’s *ρ* = − 0.33, genetic covariance = − 0.97 (SE ± 0.28)), and total sclerotia weight and relative growth at 15 °C (Pearson’s *ρ* = − 0.39, genetic covariance = − 0.63 (SE ± 0.29)). This suggests that complex trade-offs and synergisms between life history traits may influence fitness [5]. Several of the models we fit had a non-sensical genetic correlation of > 1 or < − 1. Upon further inspection, these models appeared to be over-fit to the data, since the variance components of the error terms for one or both variables were zero. Furthermore, the standard errors of the correlation estimates were extremely high (Supplementary Table 10). This may be caused by factors such as the relatively small sample size and population structure. Further, more comprehensive work is needed to fully understand the genetic covariance shared between different life history traits in *S. sclerotiorum* populations.

Several studies have shown that SVs have a major role in creating phenotypic diversity, and graph pan-genomes in which SVs can be reliably called have shown that they can be a major component of missing heritability [19, 81]. To test the impact of SVs on life history traits, we conducted two genome-wide association studies (GWASs), which we refer to as GWAS1 and GWAS2. GWAS1 included all 186,399 quality-filtered biallelic variants with a minor allele frequency of ≥ 0.05. To increase statistical power, GWAS2 used a subset of 41,843 of these variants that were in approximate linkage equilibrium. Of the original set of 5556 SVs, 2133 were biallelic, passed quality filters and had a minor allele frequency of > 0.05. Of these, 634 were in the subset used for GWAS2, 491 were in perfect LD (R^2^ = 1) with at least one of the variants used in GWAS2, and 2015 (94%) were either in the set used for GWAS2 or were in high LD (R^2^ > 0.8). The remaining 118 SVs were in lower LD with variants used in GWAS2, with R^2^ ranging from 0.2 to 0.8.

For all traits, quantile–quantile plots suggested that the model we used adequately controlled *P* value inflation due to population structure (Additional file 4: Supplementary Fig. 4). Using GWAS1, we found that the average absolute effect size of SVs was higher than that of non-SVs across 11 of the 14 traits, significantly higher for eight (*P* < 0.05), and significantly lower for 2 of the 14 traits, tebuconazole and ROS tolerance. Notably absolute effect size was on average 0.015 and 0.020 points higher for relative growth on azoxystrobin and total sclerotia weight, respectively (*P* < 0.0001, Fig. 5C, Additional file 6: Supplementary Table 11). On average, SVs had a lower average minor allele frequency than other variants (0.18 vs 0.21), which could lead to an increase in the variance of effect size estimates. Therefore, we took 500 random samples of non-SVs of equivalent size and minor allele frequency distribution to SVs and assessed how many times their average absolute effect size was more than or equal to that of the SVs. for six of the eight traits with a significantly larger effect size among SVs before correcting for allele frequency, < 0.1 of the randomisations had a mean effect size at least as large as that of the SVs. These included azoxystrobin tolerance, growth rate at 20 °C and average sclerotia weight, which were impacted more strongly by SVs than other variants in 100% of random samples (*P* < 0.002) (Fig. 5D, Additional file 6: Supplementary Table 11), and medicarpin tolerance, growth at 25 °C and total sclerotia weight, which were more strongly impacted compared with more than 90% of random samples (*P* < 0.1). According to the randomisation test, ROS and tebuconazole tolerance were also significantly more weakly impacted on average by SVs than other variants (*P* < 0.002—all randomisations had a higher mean absolute effect size). We infer from this analysis that SVs could have a larger impact on many of these traits than other variants, though genetic architecture with respect to SVs likely varies considerably between traits.

As an alternative to assessing the absolute effect size, we performed regressions on different genomic relationship matrices, which either included or did not include all variants in linkage disequilibrium with SVs. Using cross-validation, we found that for medicarpin and ROS tolerance, growth at 15 and 20 °C, and sclerotia number, models that included variants in LD with SVs had a better predictive ability than models that did not (Pearson’s *ρ* = 0.25 vs 0.23, 0.38 vs 0.37, 0.03 vs 0.02, 0.34 vs 0.29 and 0.14 vs − 0.14, respectively (Additional file 6: Additional file 17: Supplementary Table 12)). Given absolute effect size for ROS tolerance was lower on average among SVs, it is possible that although individual SVs have a relatively weak impact on this trait, as a collective they explain a relatively larger proportion of additive heritability. For the other traits, the improvement in predictive ability was in accordance with the increase in absolute effect size according to at least one of the analyses mentioned previously.

Using GWAS2, we identified 15 variants with a significant (Benjamini–Hochberg adjusted *P* < 0.05) impact on phenotype across six traits. Thirteen of these variants were intergenic SNPs or InDels, one was a synonymous SNP and the other was a disruptive in-frame InDel. The genes associated with these variants had diverse functions, which may be speculatively associated with each of the traits (Additional file 6: Additional file 18: Supplementary Table 13). Though none of these variants were SVs or in linkage disequilibrium with neighbouring SVs, one of them was a 48 bp InDel, close to the 50-bp cutoff we used for designating variants as “structural”. This variant, at position 107,298 on chromosome 12, was a deletion that significantly increased relative growth on azoxystrobin (*P* = 1.6e^−5^) (Fig. 5E). There were two genes either side of this variant on opposite strands, one encoding a 78-amino acid protein with no known domains and the other a 1516-amino acid protein containing a centrosomin domain (PF07989). The variant was, respectively, 1177 and 2571 bases away from the transcription start sites of the shorter and longer genes. The amino acid sequences of both proteins were well-conserved in fungi, with 80–100% similarity to homologues from species in the Helotiales, suggesting they are genuine genes (Additional file 6: Additional file 18: Supplementary Table 13). With the current data, it is not possible to determine which, if either, of these genes’ functions is linked with growth rate on azoxystrobin. However, in fungi, centrosomins are localised to spindle pole bodies, which are structures analogous to animal centrosomes, the main sites of coordination of microtubule activity during mitosis [82]. It is conceivable that the relative rate of cell division on a particular stressor could be impacted by mutations affecting genes encoding the cell division machinery.

Though the marker subset used for this analysis may have greater statistical power, it overlooks SVs not in linkage disequilibrium with testing markers. Both GWAS analyses miss variants with more than two alleles, which encompass more than half of the called SVs. Therefore, the absence of significant associations found for any of the SVs does not necessarily indicate that all SVs have an individually weak impact on the traits assessed.

Overall, our analyses are in accordance with several studies showing that SVs may have an outsized impact on phenotype [19, 83, 84]. In our analyses, which used a GWAS approach, we were restricted to common (minor allele frequency > 0.05), biallelic SVs. Therefore, we likely favoured less deleterious SVs, since deleterious SVs with the strongest phenotypic impacts are usually rare in populations [84, 85]. Despite this, we observed an overall stronger impact of SVs than other types of variants on many life history traits. This was also despite the observation that SV diversity tended to cluster in polymorphic, repeat-rich genome regions, which can often be sites of selective neutrality. Since the *S. sclerotiorum* genome is relatively gene-dense, containing few repeat-rich regions, this could suggest that the amount of selectively neutral structural variation it contains is relatively low. Alternatively, the variation across many of the traits we assessed could be selectively neutral or perhaps deleterious. In this case, the stronger link between SVs and phenotype is indicative of SVs underlying a particularly large amount of adaptive potential.

## Conclusion

Collectively, our results portray *S. sclerotiorum* as both a clonal and sexually outcrossing pathogen with limited diversity in gene content. Despite this limited diversity, *S. sclerotiorum* isolates vary considerably in life history traits. SVs may make a particularly strong contribution to this variability for some traits and are likely generated through two distinct mechanisms, meiotic recombination and transposition, the latter being the dominant mechanism.

The limited genic diversity of *S. sclerotiorum* contrasts the highly variable open pan-genomes of many host specialist species. Such stable gene content aligns with the hypothesis that *S. sclerotiorum*, a niche generalist, is a “jack of all trades”, with a core, multifunctional infective arsenal enabling fitness on hundreds of host species.

At this stage, the relative importance of meiosis and transposition in the generation of adaptively advantageous SVs is unknown. Given the likely general evolution of *S. sclerotiorum* towards a stable, repeat-poor genome, and the stronger contribution of SVs to variability in some life history traits, it is possible that meiosis, through its tendency to create SVs, may have a significant role in adaptation beyond its well-recognised role in recombination of alleles into new haplotypes.

Overall, our data shed considerable light on the evolutionary processes at play in an important host generalist plant pathogen of agricultural significance.

## Methods

### Assessment of life history traits

A sclerotium of each isolate was cut in half, placed on a potato dextrose agar (PDA) plate, and incubated at 20 °C for 4 to 5 days. Hyphae from the leading edge of the mycelium were cut with a 3-mm cork-borer, placed onto fresh PDA plates and incubated at 20 °C for 2 days to source actively growing mycelium. Actively growing mycelia were subcultured with a 3-mm cork-borer onto appropriate PDA plates for trait assessments.

To measure the effect of temperature on mycelium growth, mycelia were subcultured onto PDA and grown at 15, 20 and 25 °C for 1 day. To measure the effect of host metabolites on mycelium growth, mycelia were subcultured onto PDA supplemented with 50 µM brassinin (Sigma-Aldrich), 20 µM camalexin (Sigma-Aldrich), 20 µg/mL medicarpin (TargetMol) or 200 µg/mL hydrogen peroxide H_2_O_2_ (Westlab) and grown at 20 °C for 1 day. Brassinin, camalexin and medicarpin were all dissolved in DMSO prior to PDA supplementation. To measure the effect of fungicides on mycelium growth, mycelia were subcultured onto PDA supplemented with 0.2 µg/mL azoxystrobin and 50 µM salicylhydroxamic acid (SHAM), or 0.16 µg/mL tebuconazole and grown at 20 °C for 1 day. Azoxystrobin and tebuconazole were dissolved in ethanol and SHAM was dissolved in water prior to PDA supplementation. All *S. sclerotiorum* strains were also grown on PDA supplemented with the equivalent concentration of DMSO, ethanol or ethanol and SHAM as used for the aforementioned compounds. Though tebuconazole was dissolved in ethanol, ethanol control plates were not available for the experiment, so growth on tebuconazole was normalised to growth on DMSO. We did this because neither DMSO nor ethanol strongly impacted growth, whereas growth rates were often variable between experiments. For mycelium growth measurements on PDA, photographs were taken of each inoculated PDA plate. The colony area was measured using ImageJ software. Colony area relative to growth at 20 °C on PDA (plus appropriate solvent or SHAM) was calculated for each isolate.

To measure the effect of temperature on sclerotia formation, mycelia were subcultured onto PDA and grown at 20 °C for 1 month. Mature sclerotia were then air-dried for 3 days. The number and weight of sclerotia per plate were recorded.

### DNA extraction and sequencing

To extract high molecular weight DNA, sclerotia were cut with a sterile scalpel and placed with the cut side touching the surface of the medium on potato dextrose agar (PDA) plates. After 3 to 4 days at room temperature in darkness, strains were sub-cultured onto fresh PDA plates from agar plugs using a sterile cork borer and forceps. After two further days at room temperature in darkness, strains were sub-cultured again by placing four plugs for each strain into 100 ml of potato dextrose broth (PDB) in 250-ml conical flasks. These liquid cultures were incubated at room temperature with ambient light conditions in the laboratory with shaking at 150 rotations per minute (RPM) for 3 days and used to generate protoplasts.

After incubation in liquid cultures, agar plugs formed mycelium aggregates. Protoplasts were then generated by transferring two mycelium-aggregate-laden agar plugs each into 250-ml conical flasks with 40 ml enzymatic digestion solution containing 0.8 M mannitol, 200 mM citric acid/tri-sodium citrate buffer and 1.5% w/v lysing enzymes from *Trichoderma harzianum* (L1412, Sigma, now discontinued). Digestions were incubated for 3 h at 30 °C with shaking at 80 RPM. All protoplasts from each conical flask were then filtered through a 100-µm cell strainer (CLS431752, Merck) into one 50-ml falcon tube and pelleted using a swinging bucket rotor centrifuge at 1000–2000 × *g* for 2–3 min at 4 °C. Protoplast pellets were re-suspended in 200 µl Tris–EDTA (pH 8.0).

The resuspended protoplasts were then used as input for the MagAttract high molecular weight DNA extraction kit (67,563, Qiagen), which was used with the manufacturer’s protocol for blood cells with the following modifications: 80 µl proteinase K was used instead of 20 µl, 20 µl of RNase A was used instead of 4 µl, 600 µl of buffer AL was used instead of 150 µl, 25 µl of MagAttract Suspension G was used instead of 15 µl, 600 µl of buffer MB was used instead of 280 µl; before adding MagAttract Suspension G, samples were also filtered through miracloth to remove debris. High molecular weight DNA was then sequenced on an Oxford Nanopore MinION using an SQK-LSK109 library prep kit multiplexed with the native barcoding expansion pack EXP-NBD104 on a R9.4.1 version flowcell.

To extract DNA for Illumina sequencing, the same procedure was used for initial culturing of *S. sclerotiorum* strains. Cultures from PDB were then snap frozen in liquid nitrogen and freeze-dried overnight. Portions of approximately 1 g of freeze-dried samples were then cut with a sterile scalpel and placed using forceps into 2-ml screw-capped Eppendorf tubes with a single ball bearing. To each tube, 700 µl lysis buffer (50 mM Tris–HCL, 50 mM EDTA, 3% sodium dodecyl sulphate, 1% 2-mercaptoethanol) was added, and samples were ground in a MiniG model 1600 at 15,000 RPM for 2 min. Samples were then centrifuged at 17,000 RPM for 1 min, and ball bearings were removed. To each tube, 100 µl RNase A was added and tubes were then incubated for 1 h at 65 °C. To each tube, 700 µl chloroform:phenol (50:50) was added and tubes were vortexted. Tubes were then centrifuged at maximum speed for 5 min before removal of the aqueous phase. Then, 700 µl of chloroform:isamyl alcohol was added, the tubes vortexed and centrifuged again at full speed for 5 min. The aqueous phase was again removed and DNA was then precipitated using 6 M sodium acetate. Paired end Illumina sequencing was conducted at Genomics WA on a NovaSeq 6000 flowcell at 2 × 150 cycles to yield 1.2 Gb per sample. For genomic DNA methylation analysis, *S. sclerotiorum* 1980 (ATCC 18683) was propagated on minimal salts—glucose (1% w/v) (MS–Glu) agar. The inoculum for all experiments was prepared by grinding 2 g of sclerotia in 200 mL of MS–Glu in a Waring blender for 4 min. The volume was increased to 500 mL in a 1-L baffled flask and the culture incubated at 20 °C with shaking (60 r/min) for 3 days. One gram of mycelia (wet mass) was spread over a 5-cm-diameter area of *B. napus* leaf surface and incubated in a humidified chamber. Leaves from 45-day-old plants were used. Three biological replicates (3 different flasks of culture inoculated onto different leaves) were collected. The mycelial mat was collected from the lesion using forceps at 48 h post-inoculation, plant material was removed, and the samples frozen immediately in liquid nitrogen. Samples were ground in liquid nitrogen using a mortar and pestle, then genomic DNA was extracted from a 100-mg sample using the DNeasy Plant Mini kit (Qiagen). Genome Quebec performed whole genome bisulfite sequencing using the NEB Next kit, then sequenced 2 × 250 bp on an Illumina NovaSeq6000.

### Trimming and demultiplexing reads

FAST5 files from the Oxford Nanopore were basecalled using Dorado version 0.3.2 and de-multiplexed using Guppy version 6.5.7. Illumina whole genome sequencing reads were trimmed using cutadapt version 2.8 [86] with appropriate adaptor sequences. Bisulfite sequencing reads were assessed for quality and low-quality bases and adapters were trimmed using CLC genomics workbench 20.0.2.

### Analysis of bisulfite sequencing data

Methylation analysis was performed using the CLC genomics workbench 20.0.2. Reads were mapped to the *S. sclerotiorum* genome (GCF_000146945.2) using “Map Bisulfite reads” (directional mapping and default mapping options). Methylated residues were identified using “Call Methylation Levels” with default settings with the following exceptions: exhaustive context-independent calls, minimum read depth of 10 reads. The data presented in the results section is a count of methylated bases per 50-kb sliding window.

### Genome assembly

Genomes were assembled from Nanopore reads using Flye version 2.8.1-b1676 [87] and polished using Illumina reads, either from [29] or generated in this study, with one round of Polypolish version 0.5.0 [88], followed by one round of Pilon version 1.24 [89]. Before subsequent analyses, mitochondrial contigs were removed from assemblies using the following procedure. Within Geneious Prime version 21.2.2, the Minimap2 version 2.24 [90] plug-in was used to align a published *S. sclerotiorum* mitochondrial genome (NCBI accession KX351425) to each of the polished genomes. Contigs that aligned to this accession with more than 95% identity were separated from nuclear contigs, which we focus on in this study.

Polished nuclear chromosomes for each genome were scaffolded to the *S. sclerotiorum* reference genome [30] using the command ‘scaffold’, with the flags ‘-u -w -o’, from RagTag version 2.1.0 [91]. We then used the following process to finalise the scaffolded assemblies. First, Nanopore reads for each assembly were self-corrected using Canu version 2.2 [92]. Within Geneious Prime version 21.2.2, corrected reads were then aligned to their respective assemblies using the Minimap2 version 2.24 plug-in and used to manually add telomeres and subtelomeric sequences to the ends of chromosomes where they could be recovered from reads. Gaps between scaffolded contigs were also removed if there was extensive read support for joining the contigs. Read support was considered extensive if most uniquely mapped reads entirely spanned the gap and their alignment breakpoints corresponded precisely with the ends of the two contigs.

Commands from Mummerplot version 3.1 [93] were then used to check for misassemblies. First, ‘nucmer’ was used to align each assembly individually to the *S. sclerotiorum* reference genome with the option ‘-mum’. The ‘delta-filter’ command was then used, with the options ‘ -1 -i 95 -l 10,000 -u 100’ to filter the output of nucmer. The filtered output was then passed to the command ‘show-coords’ to produce coordinates of scaffold mappings to the *S. sclerotiorum* reference genome. The command ‘awk 'NR > 5 {strand = " + "; if($2 < $1){strand = "-"};print $12"\t"$1"\t"$2"\t"$13"\t.\t"strand}'’ was then used to convert these coordinates into browser extensible data (BED) format. In Geneious, the BED file containing alignments to 1980 and mappings of self-corrected reads were used to judge whether chromosome segments had been artificially joined by the assembler. For instance, if one chromosome was unusually large and contained two different segments mapped to different 1980 chromosomes, it was split in two if (i) very few reads supported the join and (ii) reads showed evidence of extensive soft clipping either side of the join. Where chromosomes were split, genomes were scaffolded a second time using RagTag and gaps between joined chromosome segments were removed if aligned reads supported the join. The majority of chromosomes had zero gaps after the first round of scaffolding and only two in each of three strains were broken and re-scaffolded based on the latter procedure.

### Genome annotation

For comparative purposes, all genomes, including the reference genome, were annotated with the same procedure. First, repetitive sequences were annotated using EDTA version 2.2 [94] with the flag ‘--anno 1’. Then, Braker3 [95] was used to annotate genes with both RNA sequencing and amino acid sequences as evidence with the additional flags ‘--fungus’, ‘--prot_seq = Fungi.fa’, ‘--august_args = ”--species = botrytis_cinerea”’. The RNA sequencing data used for annotation were derived from 32 samples from the sequence read archive (SRA) detailed in Additional file 6: Additional file 19: Supplementary Table 14. Reads from these samples that were derived from infected plant tissue were first filtered by alignment to their respective host genomes (Additional file 6: Additional file 19: Supplementary Table 14) with Hisat2 version 2.1.0 [96] and keeping unmapped reads with ‘--un-conc’ for paired end reads or ‘--un’ for single end reads. Filtered reads, and reads not from plants, were then aligned to each of the *S. sclerotiorum* genomes with Hisat2, converted to bam format with samtools version 1.10 [97] ‘view’ and used as input for Braker3. Amino acid sequences from Braker3 annotations were combined into a non-redundant set of genes for all isolates using cd-hit version 4.8.1 [98], and non-redundant proteins were annotated with InterProScan version 5.54–87.0 [99]. Secondary metabolite clusters in this set of proteins were identified using antiSMASH version 7.0 [100], and secreted proteins were identified with SignalP version 6.0 [101].

### Pan-genome graph construction and variant calling

Using the 24 Nanopore assemblies and the *S. sclerotiorum* reference genome (GenBank reference GCA_001857865.1), a pan-genome graph genome was constructed with cactus 2.5.2 [102]. Illumina reads from [29] and those generated in the current study were mapped to the pan-genome GBZ formatted graph using the ‘giraffe’ command of vg version 1.52.0 [18]. The resulting GAM formatted files, one for each set of Illumina reads, were filtered using the vg command ‘filter’, with the flags ‘--min-primary 0.90 --frac-score --substitutions --min-end-matches 1 --min-mapq 15 --defray-ends 999’. Filtered GAM files were then passed to the vg command ‘pack’ to create pack formatted read support files for each variant, with the flag ‘--min-mapq 5’. The ‘call’ command from vg was used to call variants from the pan-genome graph using the Illumina reads with the flags ‘--ploidy 1 --genotype-snarls’ and create a variant call format (VCF) file. The VCF files for all samples were combined into a single file by first converting them to gzip format with ‘bgzip’, then merging them with the bcftools version 1.10.1 [103] command ‘merge’, with the option ‘--all’. We then filtered this VCF with vcftools version 0.1.16 with the options ‘--minQ 30’ and ‘--minDP 5’. To do this, we had to first set all variants to ‘PASS’ because bcftools merge adds a filter to the whole variant if only a single sample is filtered in one of the inputs. We did this using a simple Awk script.

After calling variants present in the pan-genome graph, additional variants present in Illumina reads but not in the 25 genomes that made up this graph were called using the following procedure. First, filtered GAM files were converted to binary alignment map (BAM) files using the vg command ‘surject’ and sorted using the samtools command ‘sort’. Then, the command ‘mpileup’ from bcftools was used with the flags ‘--max-depth 1000 --output-type u’ and the BAM files as input. The output of ‘mpileup’ was piped to the bcftools command ‘call’, which was run with the flags ‘--output-type v --multiallelic-caller --ploidy 1’ to create a VCF file. We then filtered this VCF using vcftools with the options ‘--minQ 30’, ‘--minGQ 30’ and ‘--minDP 5’.

Finally, we used vcftools to remove variants called by vg from the VCF created using bcftools with the options ‘--min-alleles 2’, ‘--mac 1’ and ‘--exclude-positions’, and concatenated the resulting VCF with the one produced using vg with the bcftools command ‘concat’ with the option ‘--allow-overlaps’. We further filtered the final VCF with a Python script (Additional file 21: Supplementary File 2) to remove variants with a missing call rate of ≥ 0.2.

### Population structure characterisation

To identify clones, a VCF containing variants called against the graph pan-genome was used with plink version 1.9 [104] to generate an identical by state relationship matrix, with the flags ‘--snps-only’, ‘--biallelic-only’, ‘--double-id’, ‘--geno 0.2’, ‘--mind 0.2’ and ‘--make-rel square 1-ibs’. Then, the matrix was used to construct a distance matrix and dendrogram using hierarchical clustering. Clones were identified based on a relatedness of 98% identical by state with the R base function ‘cutree’. This threshold was arbitrarily chosen to divide the strains into groups that contained individuals whose pairwise identities were no less than 99.82% and those that were a maximum of 98% identical. There were no groups between these two extremes. We chose this threshold as it clearly delineated groups of nearly identical strains from more deeply diverging, likely recombinant, lineages (Additional file 1: Supplementary Fig. 1). To produce the phylogenetic network in Fig. 1, we used the R package ‘phangorn’ version 2.11.1.

Population structure was analysed using ADMIXTURE version 1.3 [105]. As for the identical by state relationship matrix, we considered only biallelic SNPs. These were first filtered using plink with the flag ‘--indep-pairwise 50 10 0.1’, and admixture was run for 1 to 10 ancestral populations with cross-validation. A scree plot was used to determine the most appropriate number of populations to use based on cross-validation error (Additional file 5: Supplementary Fig. 5). Principal component analysis was also performed with plink using the flag ‘--pca 4’.

### Assessment of structural variant diversity

To assess the diversity of SVs across the genome, we developed a novel statistic that we refer to as ${SV}_{\pi }$, which is calculated as follows. First, we calculate ${SV}_{n}$, which is the sum of the number of SVs between all pairs of individuals, excluding self-comparisons.

$${SV}_{n}= \sum_{i=1}^{n-1}\sum_{j=i+1}^{n}{SV}_{ij}$$

where ${SV}_{ij}$ is the number of variants that are ≥ 50 bp in at least one individual for individuals $i$ and $j$ in the set of $n$ individuals in the sample. ${SV}_{n}$ is then normalised in the following way to obtain ${SV}_{\pi }$:

$${SV}_{\pi }= \frac{{SV}_{n}}{n(n-1)/2} \div L$$

This divides ${SV}_{n}$ by the number of possible pairs of individuals and the length of the sequence under consideration, $L$. Since $L$ varies between individuals depending on the SV alleles they contain, it is calculated in the following way:

$$\frac{1}{n}\sum_{i=1}^{n}{k}_{i}$$

That is, $L$ is the average value of all $n$ sequence lengths in the vector of sequence lengths $k$, in kb. The statistic is trivial for regions containing only biallelic SVs but more computationally challenging for regions with multi-allelic variants.

The statistic ${SV}_{\pi }$ is an estimate of the average number of SVs that are present per kb between all pairs of individuals in the sample. It is an approximation of the genome stability in a region and may be better at identifying unstable genomic regions than considering simpler statistics such as proportion of rearranged sites or number of SVs relative to a single reference. The reason we developed this statistic was because we aimed to better capture the potential evolutionary rate of a region. For example, if considering the fraction of non-syntenic bases, a single large variant would create a high value, even if it is the only variant present. On the contrary, many diverse, small SVs would possibly cause a deflated estimate of the SV diversity of the region if their total length was a small proportion of the region’s overall length. Though we do not present a detailed exposition of the method here, we present it as an intuitive and hopefully useful complementary technique for investigating structural diversity in pan-genomes. Our software for its calculation across sliding windows, svstats, is freely available on GitHub (https://github.com/markcharder/svstats). We used the program in this study to calculate SVπ in 50 kb sliding windows across the genome with an increment of 1 kb.

### Analysis of linkage disequilibrium and recombination

To assess linkage disequilibrium decay with physical distance, linkage disequilibrium was first calculated for all pairs of variants between variants with the plink flags ‘--ld-window-r2 0’, ‘--ld-window-kb 300’ and ‘--r2 dprime’. R^2^ was averaged for each physical distance and the distance at which average R^2^ reached half its maximum value was recorded. The programme phipack (obtained from https://www.maths.otago.ac.nz/~dbryant/software/PhiPack.tar.gz) was used to conduct three tests of the association between distance and linkage disequilibrium, the pairwise homoplasy index, maximum Χ^2^ and nearest neighbour score tests.

To assess recombination rate, we selected four genotypically fairly uniform populations that had no obvious population structure. This was done by manually selecting non-admixed and non-clonal individuals from the same putative ancestral population identified with ADMIXTURE. The strains included in each of the populations are identified in Additional file 7: Supplementary Table 2. Overall, there were four populations. Populations 1, 2, 3 and 4 contained 22, 13, 15 and 34 individuals, respectively. Since many individuals from both North America and Europe had the same ancestry, we either considered Canadian samples separately (population 3) or samples from both regions together (population 4). Recombination rate was calculated for these populations using ldhat version 2.2 [57] and recombination hotspots were identified with ldhot version 8.30 [106]. To run ldhat and ldhot, plink was first used to convert the pan-genome VCF file to plink PED and MAP files with the flags ‘--recode’, and ‘--biallelic-only’ and ‘--snps-only’ to keep only biallelic SNPs. These were used as input for the command ‘plink2ldhat’ from our programme ‘svstats’ (https://github.com/markcharder/svstats) to convert to ldhat or ldhot format. A finite sites version of Watterson’s theta [107] was calculated using the command ‘watfsites’ from svstats to provide a parameter for generating ldhat lookup tables. The ldhat programme ‘complete’ was then used, with flags ‘-rhomax 100’ and ‘-n_pts 101’, and the appropriate number of ‘-n’ individuals, to create look-up tables for calculating variable recombination rates across chromosomes. The ldhat interval programme was then used, with the appropriate look-up table, to calculate variable recombination rates with the flags ‘-exact’ ‘-its 10,000,000’ and ‘-samp 3500’. Reversible jump Monte Carlo Markov Chains were run starting with block penalties ranging from 5 to 50 (with an increment of 5) and chains were assessed for convergence. Posterior distributions of rates and bounds from the chains were estimated using the ldhat command ‘stat’, with the flag ‘-burn 35’. Using the output of ldhat interval, ldhot was run using the appropriate look-up table with the additional flag ‘--nsim 1000’.

### Assessment of correlation between population-wide statistics and genomic features

The command ‘makewindows’ from Bedtools version 2.27.1 [107] was used to create sliding windows of 50,000 bp, with an increment of 1000 bp, across the *S. sclerotiorum* genome. To calculate gene and repeat density, the bedtools command ‘coverage’ was used with Braker3 and EDTA annotations, respectively, and the sliding windows. To count the number of SVs, the same module was used with the flag ‘-counts’. To calculate GC content for windows, the command ‘nuc’ was used. Methylation rates from bisulfite sequencing data were converted to BED format using a custom script in R, and bedtools ‘intersect’ with the flag ‘-c’ was used to calculate the number of methylated sites per sliding window. A BED file was also created from the ldhat output, using a simple Awk script, and used to calculate recombination rate for sites in sliding windows. The rate was summed across sliding windows for comparison. Comparison between recombination rate and other statistics of interest was conducted in R using Spearman’s rank correlation.

Using the bedtools module ‘interval’, we labelled sliding windows as ‘hotspots’ if they overlapped a hotspot identified using ldhot and ‘non-hotspots’ if they did not. We then compared statistics including number of SVs, SVπ, percent repeat, and percent gene between non-hotspot and hotspot windows using Welch’s *t* tests. To increase sensitivity of hotspot detection, we combined hotspots across all four populations using the bedtools module ‘merge’ with a concatenation of BED files containing sliding windows overlapping hotspots. We present both the comparisons made using the merged hotspots (referred to as the ‘union’) and for hotspots in each individual population.

To complement the results of the *t*-tests, we also conducted randomisation tests. For these tests, 1000 samples of loci corresponding in size to hotspot regions were created using the svstats module ‘randcoords’. Gene and repeat coverage, and SV counts were calculated as mentioned above using bedtools for random coordinates and the hotspot coordinates. We also recorded SVπ for all 50-kb windows overlapping at least one hotspot or random coordinate. We then took the average of these statistics for each randomisation and calculated *P* values by taking the fraction of randomisations where the value was below (for genes and repeats), above (for SVπ and SV counts) or equal to the average for the hotspots. This was performed for all populations individually and for the union of all hotspots across all populations.

The bedtools command ‘closest’ was used to determine the distance between transposon annotations from EDTA and the nearest structural variant in all genomes. A Kruskal–Wallis test in R was then used to determine whether any transposon classes were significantly closer than other classes to the nearest SV. To assess pairwise differences between classes, Dunn’s post hoc test was used. The Kruskal–Wallis and Dunn’s tests were performed using the R package ‘dunn.test’ version 1.3.6.

To determine whether SVs were significantly closer to transposons than randomised loci, we used the following procedure. We first produced a set of random SV coordinates for each strain, by randomising SV start and end coordinates whilst maintaining SV length. We then recorded the distance of these random coordinates to the nearest transposon using the bedtools ‘closest’ module, with the settings ‘-d -t first’. We did this 1000 times for each strain, and each time took the average distance across all strains. The values from the 1000 randomisations were compared with the average distance between real SV loci and transposons across all strains. The procedure for randomising loci based on a BED formatted file is include in the svstats software package we developed as a part of this study (https://github.com/markcharder/svstats).

### Genome-wide association and trait correlation analyses

Before conducting GWAS and whole genome regression analyses, phenotype data were normalised with the R package bestNormalize version 3.5. We only considered biallelic variants with a minor allele frequency of ≥ 0.05. Two GWASs were run. GWAS1 used all variants and GWAS2 used variants that were filtered so that they were in approximate linkage equilibrium using Plink version 1.9 with the flag ‘--indep-pairwise 50 kb 50 0.8’. Both GWASs were conducted using GAPIT [108] with the BLINK model. The motivation for development of models like BLINK is that causal variants may be correlated with population structure, and a kinship matrix fit as a random effect can reduce sensitivity for detecting such variants [109, 110]. Adding a kinship matrix to these models re-introduces the original confounding factor. We therefore did not use a kinship matrix. There were no non-genetic confounding factors between the populations we assessed as phenotypic data were collected in the same environment. We therefore included no further population structure correction with, for example, principal components or a kinship matrix. GWAS2 was used to identify significant marker trait associations as it had fewer correlated markers than GWAS1 and therefore more statistical power. GWAS1 was used for the comparison of average absolute effects from structural and non-structural variants.

To determine whether SVs had a larger impact on traits than other variants, we conducted three tests. Firstly, we simply used standard *t* tests to compare the mean distributions of absolute effect sizes of non-SVs and SVs. Since SVs had a lower minor allele frequency on average than non-SVs, and this could affect variance of the test statistic, we used a randomisation test. This test sampled non-SVs 500 times, each time creating a random set of non-SVs matching in number the total count of SVs. This random set was sampled so that proportions of variants with all possible minor allele frequencies (rounded to three significant digits) matched the minor allele frequency proportions in the SV set. The average absolute effect size from GWAS2 was recorded for each of these 500 samples and the fraction of times this effect size was larger than or equal to that of the mean absolute effect size of the SVs was treated as the empirical *P* value.

In our third test, we partitioned variants into those that were in linkage disequilibrium with structural variants and those that were not. We did this by first creating a file recording R^2^ for all pairs of neighbouring variants within 2 kb with the plink flags ‘--r2’ and ‘--ld-window-kb’. From this file, we created a list of variants that had an R^2^ of ≥ 0.5 with at least one SV. This list, combined with the list of SVs themselves, was used to create two VCF files, one containing SVs and variants in approximate linkage disequilibrium with them and the other containing variants that were not SVs and were not in linkage disequilibrium with any SVs. The two VCFs were filtered so that variants were not in strong linkage disequilibrium with the plink command ‘--indep-pairwise 50 kb 50 0.8’. Genomic relationship matrices [111] were created for each of these sets of variants and for the whole set of variants used in GWAS1 with the plink flag ‘--make-rel square’.

To assess genetic correlations between traits and determine whether adding SVs improved predictive ability, we fit univariate and multivariate linear mixed models with the R package sommer version 4.3.4 [112]. In these models, random effects for individuals were estimated with assumed variance and covariance described by the genomic relationship matrix proposed by Yang et al. [111]. To assess trait genetic correlations, the genomic relationship matrix was estimated using all biallelic variants of minor allele frequency ≥ 0.05. To assess improvement of prediction accuracy when including SVs, we fit models with one random effect with variance structured by a relationship matrix estimated with only non-SVs, and models with two random effects, one structured by a non-SV and the other structured by an SV-only relationship matrix. For each trait, we performed ‘leave-one-out’ cross validation. For each recording of each phenotype for each strain, the recording was masked and the two models fit with this recording missing. The predicted BLUP value based on the rest of the strains was recorded for this version of the model and Pearson’s correlation coefficient between model predictions and phenotypes was recorded.

## Supplementary Information

Additional file 1: Supplementary Figure 1. A dendrogram showing the percentage of alleles identical by state between strains in the collection. The green vertical line shows the cutoff used to identify groups of individuals representing a single clone (blue).

Additional file 2: Supplementary Figure 2. The relationship between recombination rate (y axis) and coding sequence density (x axis) of 50 Kb sliding windows. The line is a general additive model and the shading represents 95 % confidence intervals.

Additional file 3: Supplementary Figure 3. SVπ and repeat content in 50 Kb windows across the genome. The same as Figure 3 D but shown for all chromosomes.

Additional file 4: Supplementary Figure 4. Q-Q plots for GWASs conducted for all traits. The y axis shows observed P values and the x axis shows the expected P values given a normal distribution. All plots show that most points are on (adequate correction) or below (over-correction in some cases) the line, and P values are not inflated.

Additional file 5: Supplementary Figure 5. Scree plot showing cross-validation error of different numbers of k populations tested with ADMIXTURE. The lowest cross-validation error was for k = 6 populations, so this was the number chosen to model population structure.

Additional file 6: Supplementary Table 1. A BUSCO scores for all strains used to construct the pan-genome graph. B. Gaps and telomeres in each chromosome of each assembly. In the TELOMERES column, L stands for ‘left’ and R stands for ‘right’, referring to the two (arbitrary) ends of the chromosome in the assembly FASTA.

Additional file 7: Supplementary Table 2. Strains, excluding the reference strain, 1980, used to create the *Sclerotinia sclerotiorum* pan-genome and call structural variants. Strains with Nanopore and Illumina data were used to create the pan-genome graph whereas strains with only Illumina data (previous or current study) were used for mapping and variant calling against the graph.

Additional file 8: Supplementary Table 3. Results of phipack tests for recombination across the 120 independent *Sclerotinia sclerotiorum* lineages. These include the Neighbour Similarity Score (NSS), the Maximum Chi^2 (MAX_CHI2), and the Pairwise Homoplasy Index (PHI) tests. All tests were significant, with a P value of zero, indicating increasing levels of recombination between alleles with distance.

Additional file 9: Supplementary Table 4. A Spearman's rank correlation between distance from chromosome end and estimated recombination rate across the four population sub-samples and chromosomes (summarised in Figure 2 B). Rows highlighted in orange show a negative correlation and in green a positive. Grey rows were not significant. In most instances, there was a significant negative correlation between recombination rate and distance from chromosome end. B Recombination hotspots identified relative to the *Sclerotinia sclerotiorum* reference genome. Four non-structured population subsamples were used to identify hotspots.

Additional file 10: Supplementary Table 5. Sheets 1-6 are Cytosine methylation data from alignment of bisulfite sequencing reads to the 1980 genome. The first column is the NCBI chromosome accession. The columns for these tables are described in the CLC genomic workbench manual here: https://resources.qiagenbioinformatics.com/manuals/clcgenomicsworkbench/current/index.php?manual=Call_Methylation_Levels.html. Each spreadsheet represents one of the samples, for example, 0 HPI R1 is 0 hours post-inoculation replicate 1. Sheet 7 (labelled'hotspot t-tests') is the results of t-tests to assess the difference between windows containing, or not containing, recombination hotspots, for gene density, number of structural variants, repeat density and SVπ.

Additional file 11: Supplementary Table 6. Results of Dunn’s post hoc test to determine whether LTR transposons were significantly closer to structural variants across all genomes than other transposons. Transposon classifications are taken from EDTA.

Additional file 12: Supplementary Table 7. Transposable element content of the 24 *Sclerotinia sclerotiorum* genomes based on EDTA annotations.

Additional file 13: Supplementary Table 8. Spearman’s correlation between estimated recombination rate and SVπ, SV count and transposon content of 50,000 bp sliding windows. Rows coloured in green are significant positive correlations, those in red are significant negative correlations and those not coloured are not significant. Overall, the majority of chromosomes and populations showed a correlation between recombination rate and both SVπ and SV count but not transposon content.

Additional file 14: Supplementary Table 9. Functional terms associated with genes in the largest gene bubble. Results are from an InterProScan analysis. The Gene IDs are based on a cd-hit grouping of Braker3 annotations across all genomes.

Additional file 15: Supplementary Table 10. Genetic and actual correlations between life history traits. Where genetic correlations are above 1, below -1 or ‘NA’, the model was likely poorly or over-fit.

Additional file 16: Supplementary Table 11. Tests for overall impact of SVs on phenotype. Grey cells are for test statistics that were not significant. Green cells are for test statistics that indicate in increase in SV impact on phenotype. Red cells are for test statistics that indicate a decrease in SV impact on phenotype.

Additional file 17: Supplementary Table 12. Linear mixed models testing improvement in predictive ability (Pearson’s ρ) from models with no SVs in the genomic relationship to matrix to models with two terms, one for SVs and the other for non-SVs, or to models with only SVs. Improvements in predictive ability were variable but some traits showed a relatively large improvement.

Additional file 18: Supplementary Table 13. A Results of a GWAS for 14 life history traits. B BLASTp hits for gene downstream of 48 bp InDel azoxystrobin QTL, which encodes a centrosomin. C BLASTp hits for gene upstream of 48 bp InDel azoxystrobin QTL, which encodes a protein with no known functional domains.

Additional file 19: Supplementary Table 14. A RNA sequencing data used for Braker3 annotation of genomes. B The host genomes used for filtering RNA sequencing reads used in Braker3 annotation.

Additional file 20: Supplementary File 1. Plots showing the same data as Figure 2 H for all populations and chromosomes.

Additional file 21: Supplementary File 2. Python script used to remove variants from a VCF with a missing call rate of >= 0.2.

## Data Availability

The datasets generated and/or analysed during the current study are available in NCBI, under BioProjects PRJNA1112094 and PRJNA1120954, in the supplementary material, or available upon reasonable request from the corresponding author.

## Abbreviations

PCA: Principal component analysis
GWAS: Genome-wide association study
BLUP: Best linear unbiased predictor
SV: Structural variant
InDel: Insertion/deletion
SNP: Single-nucleotide polymorphism
MNP: Multiple nucleotide polymorphism
DNA: Deoxyribonucleic acid
RNA: Ribonucleic acid
LTR: Long terminal repeat
VCF: Variant call format
LD: Linkage disequilibrium
