# Supplementary figures and images for: Recombination and transposition drive genomic structural variation potentially impacting life history traits in a host-generalist fungal plant pathogen
Source: BMC Biol. 2025 Apr 28;23:110. doi: 10.1186/s12915-025-02179-x (PMC12036203; doi:10.1186/s12915-025-02179-x)

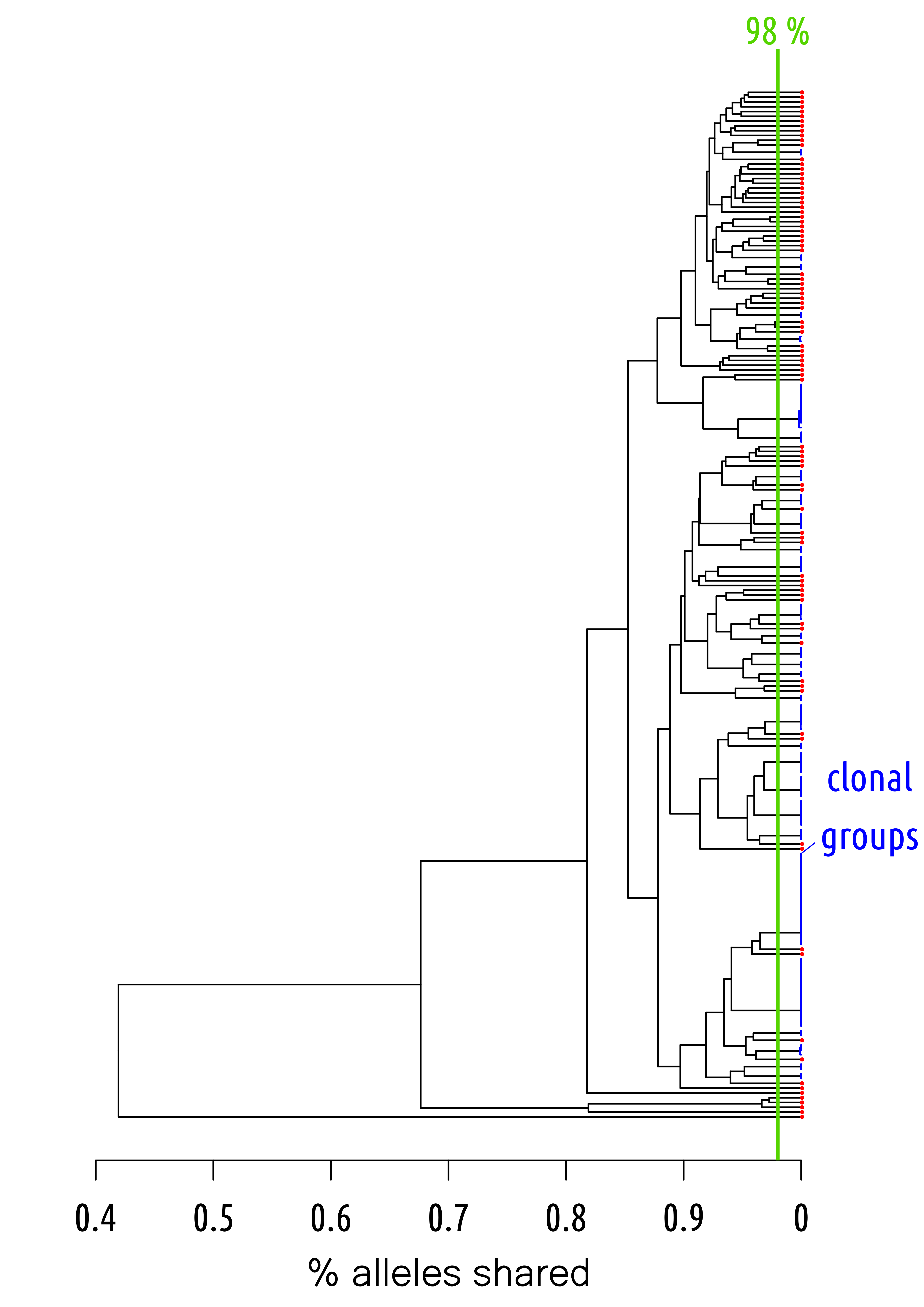

Supplement: Supplementary file 1 — Additional file 1: Supplementary Figure 1. A dendrogram showing the percentage of alleles identical by state between strains in the collection. The green vertical line shows the cutoff used to identify groups of individuals representing a single clone (blue). [file 12915_2025_2179_MOESM1_ESM.png]

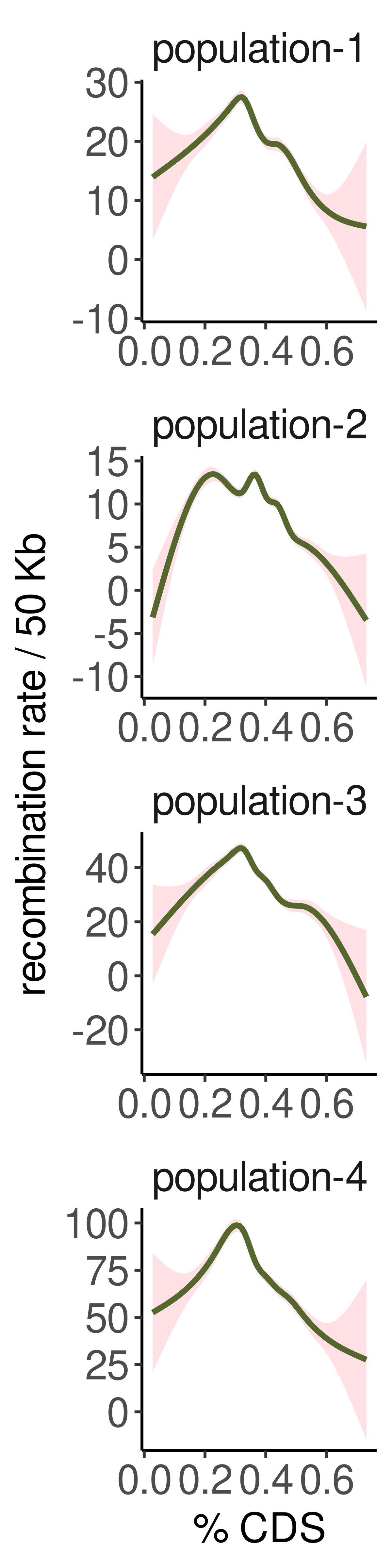

Supplement: Supplementary file 2 — Additional file 2: Supplementary Figure 2. The relationship between recombination rate (y axis) and coding sequence density (x axis) of 50 Kb sliding windows. The line is a general additive model and the shading represents 95 % confidence intervals. [file 12915_2025_2179_MOESM2_ESM.png]

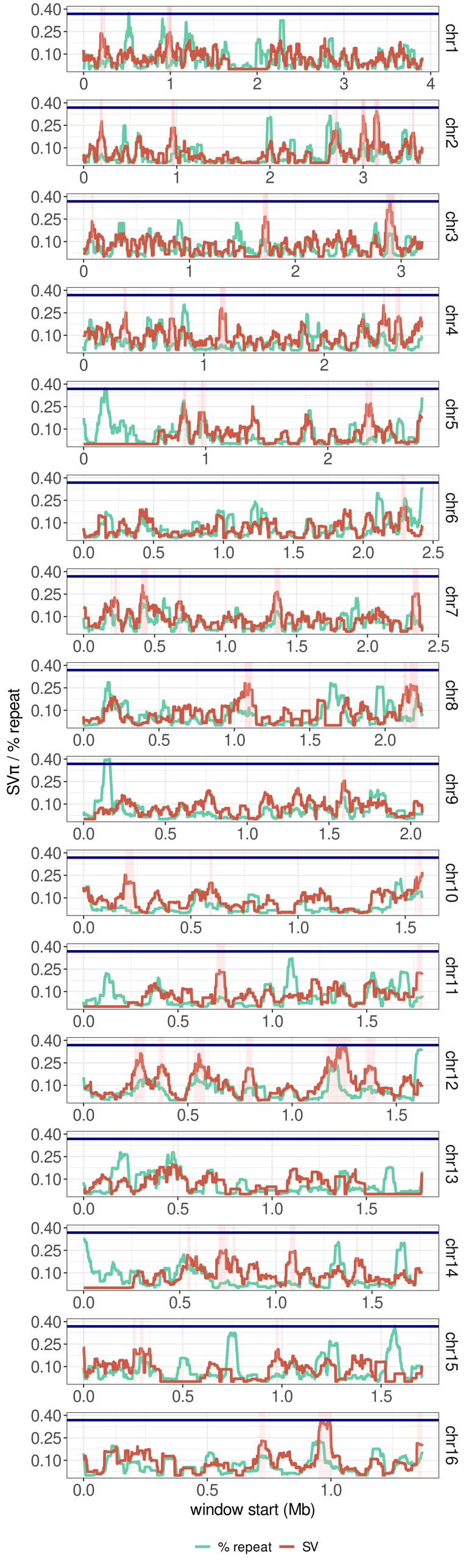

Supplement: Supplementary file 3 — Additional file 3: Supplementary Figure 3. SVπ and repeat content in 50 Kb windows across the genome. The same as Figure 3 D but shown for all chromosomes. [file 12915_2025_2179_MOESM3_ESM.png]

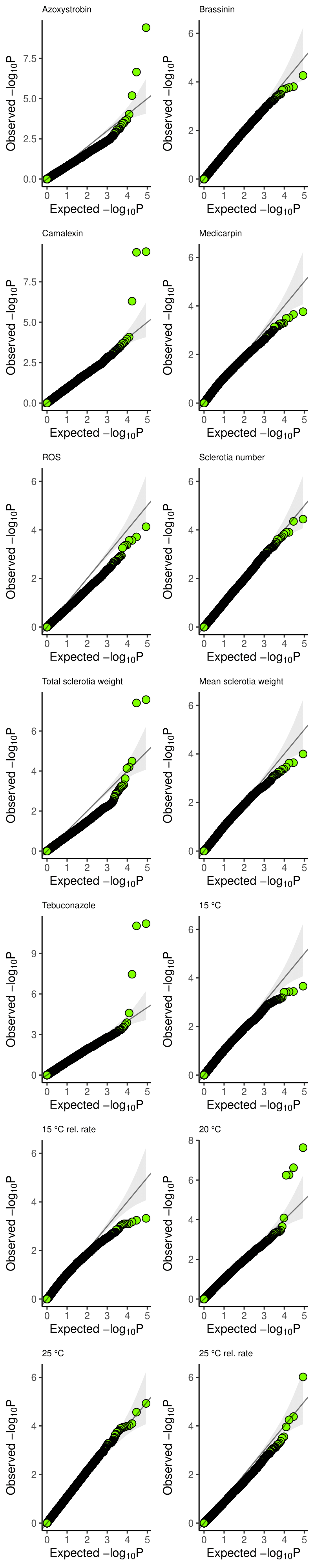

Supplement: Supplementary file 4 — Additional file 4: Supplementary Figure 4. Q-Q plots for GWASs conducted for all traits. The y axis shows observed P values and the x axis shows the expected P values given a normal distribution. All plots show that most points are on (adequate correction) or below (over-correction in some cases) the line, and P values are not inflated. [file 12915_2025_2179_MOESM4_ESM.png]

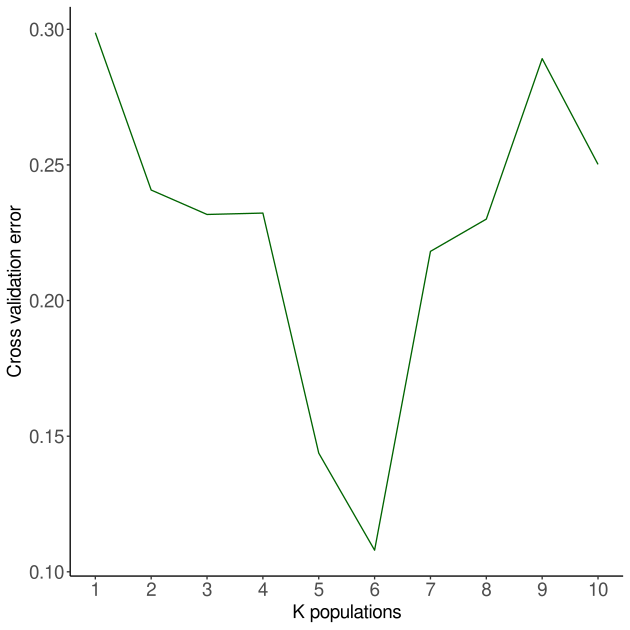

Supplement: Supplementary file 5 — Additional file 5: Supplementary Figure 5. Scree plot showing cross-validation error of different numbers of k populations tested with ADMIXTURE. The lowest cross-validation error was for k = 6 populations, so this was the number chosen to model population structure. [file 12915_2025_2179_MOESM5_ESM.png]
